# Using vaccine Immunostimulation/Immunodynamic modelling methods to inform vaccine dose decision-making

**DOI:** 10.1038/s41541-018-0075-3

**Published:** 2018-09-17

**Authors:** Sophie J. Rhodes, Jeremie Guedj, Helen A. Fletcher, Thomas Lindenstrøm, Thomas J. Scriba, Thomas G. Evans, Gwenan M. Knight, Richard G. White

**Affiliations:** 1 TB Modelling Group, CMMID, TB Centre, London School of Hygiene and Tropical Medicine, London, UK; 2 IAME, UMR 1137, INSERM, Université Paris Diderot, Sorbonne Paris Cité Paris, France; 3 Univ Paris Diderot, Sorbonne Paris Cité, F-75018 Paris, France; 4 Immunology and Infection Department, London School of Hygiene and Tropical Medicine, London, UK; 5 Statens Serum Institut, Copenhagen, Denmark; 6 South African Tuberculosis Vaccine Initiative, Institute of Infectious Disease and Molecular Medicine and Division of Immunology, Department of Pathology, University of Cape Town, Cape Town, South Africa; 7 Vaccitech, Oxford, UK

## Abstract

Unlike drug dose optimisation, mathematical modelling has not been applied to vaccine dose finding. We applied a novel Immunostimulation/Immunodynamic mathematical modelling framework to translate multi-dose TB vaccine immune responses from mice, to predict most immunogenic dose in humans. Data were previously collected on IFN-γ secreting CD4+ T cells over time for novel TB vaccines H56 and H1 adjuvanted with IC31 in mice (1 dose groups (0.1–1.5 and 15 μg H56 + IC31), 45 mice) and humans (1 dose (50 μg H56/H1 + IC31), 18 humans). A two-compartment mathematical model, describing the dynamics of the post-vaccination IFN-γ T cell response, was fitted to mouse and human data, separately, using nonlinear mixed effects methods. We used these fitted models and a vaccine dose allometric scaling assumption, to predict the most immunogenic human dose. Based on the changes in model parameters by mouse H56 + IC31 dose and by varying the H56 dose allometric scaling factor between mouse and humans, we established that, at a late time point (224 days) doses of 0.8–8 μg H56 + IC31 in humans may be the most immunogenic. A 0.8–8 μg of H-series TB vaccines in humans, may be as, or more, immunogenic, as larger doses. The Immunostimulation/Immunodynamic mathematical modelling framework is a novel, and potentially revolutionary tool, to predict most immunogenic vaccine doses, and accelerate vaccine development.

## Mathematical modelling: TB vaccine responses

Pharmacodynamic mathematical modelling has long-been successfully applied to optimise drug dosing but has not been widely leveraged for vaccine regimens. Richard G. White and colleagues fit a novel mathematical model to mouse vaccine responses elicited by the experimental TB subunit vaccine H56 adjuvanted with IC31. The authors use interferon-γ frequency in CD4^+^ T cells as arguably the most relevant single parameter in TB responses. Responses were peaked not saturating, suggesting that optimal responses might occur at lower doses of vaccine. Indeed, the analysis suggests that relatively low doses (0.8–8 μg) of H-series vaccines (H1 and H56) in IC31 might in fact be the most immunogenic at least at late time points. Although the conclusions await clinical validation, the novel modelling approach used here could in principle be applied to optimise dosing of almost any vaccine.

## Introduction

Vaccines are one of the most effective interventions in public health.^1^ However, to progress a vaccine from discovery to licensure can take decades and cost up to US$0.8 billion.^2^ With costs so high, it is vital that development is made more efficient. A primary goal in vaccine development is to establish optimal vaccine efficacy, and vaccine dose amount (hereafter ‘dose’) is a crucial factor in achieving this. The consequences of selecting the wrong dose can lead to inadequate protection against disease, and ultimately wasted resources and lives.

In humans, vaccine dose decisions are made based on dose escalation trials, the dose range of which is based on experiments in animals. In classical pre-clinical experiments, an initial dose is tested and incrementally increased until the dose is no longer considered safe. The resulting maximum safe dose is then scaled-up to be applied in a clinical setting. Historically, pre-clinical dose escalation experiments assume the response ‘saturates’, i.e. increases, then plateaus, as vaccine dose is increased. Many vaccines have progressed through developmental phases with doses selected under this assumption.^3,4^

However, recent pre-clinical data suggest that this ‘saturating’ assumption may not always be correct. Studies in mice,^5^ and humans,^6^ using the potential tuberculosis (TB) vaccine H4 adjuvanted with IC31^®^ (H4 + IC31) have shown that lower vaccine doses have higher immunogenicity and protective efficacy than higher doses. We have recently shown that the IFN-γ dose–response curve in mice, for the novel TB vaccine H56 + IC31, was peaked, not saturating,^7^ and an ongoing phase 1/2a H56 + IC31 dose-ranging clinical trial will test this prediction in humans (ClinicalTrials.gov No. NCT01865487). Similar non-saturating dose–response curves have been observed in clinical trials in HIV and Malaria vaccines using other adjuvants.^8,9^ These data suggest that developing vaccines based on a ‘saturating dose’ response curve assumption is likely to lead to sub-immunogenic doses being selected for later stage vaccine development, and risk efficacious vaccine discovery.

In contrast to vaccine development, drug development benefits from systematic, quantitative analysis through the application of Pharmacokinetic/Pharmacodynamic (PK/PD) modelling. PK/PD modelling employs mechanistic mathematical models to quantify drug concentration dynamics in the host over time (PK) and drug effect as the concentration varies (PD).^10^ Model-based drug development (MBDD) is recognised as an efficient tool to accelerate and streamline drug development, by minimising developmental time and resources.^11^ MBDD has been established for decades in the pharmaceutical industry^12^ and is often required by regulatory agencies in all stages of drug development. As such, MBDD is regularly used to establish optimal drug dose^13^ and translate drug response dynamics between species.^14^

PK/PD model-based methods have not been applied in vaccine development for dose decision making.^1^ The application of quantitative methods similar to that of MBDD, could lead to better evaluation and translation of the vaccine dose–response data from animals to humans, and accelerate vaccine development.

Consequently, we propose the novel vaccine *Immunostimulation/Immunodynamic (IS/ID) modelling framework* as a method to inform vaccine dose decision making. Analogous to PK/PD modelling, IS/ID modelling applies mathematical models to describe the underlying mechanisms, the immune response stimulation (IS) that produce the measured immune response dynamics following vaccination (ID). Like PK/PD modelling, these models are fitted to data using established statistical frameworks. Mathematical models representing the immune response to infection and vaccination, that could be considered suitable IS/ID models exist (e.g. refs.^15–17^), but up until now, no such models have been incorporated into a PK/PD style framework to inform vaccine dose prediction.

**Fig. 1:**
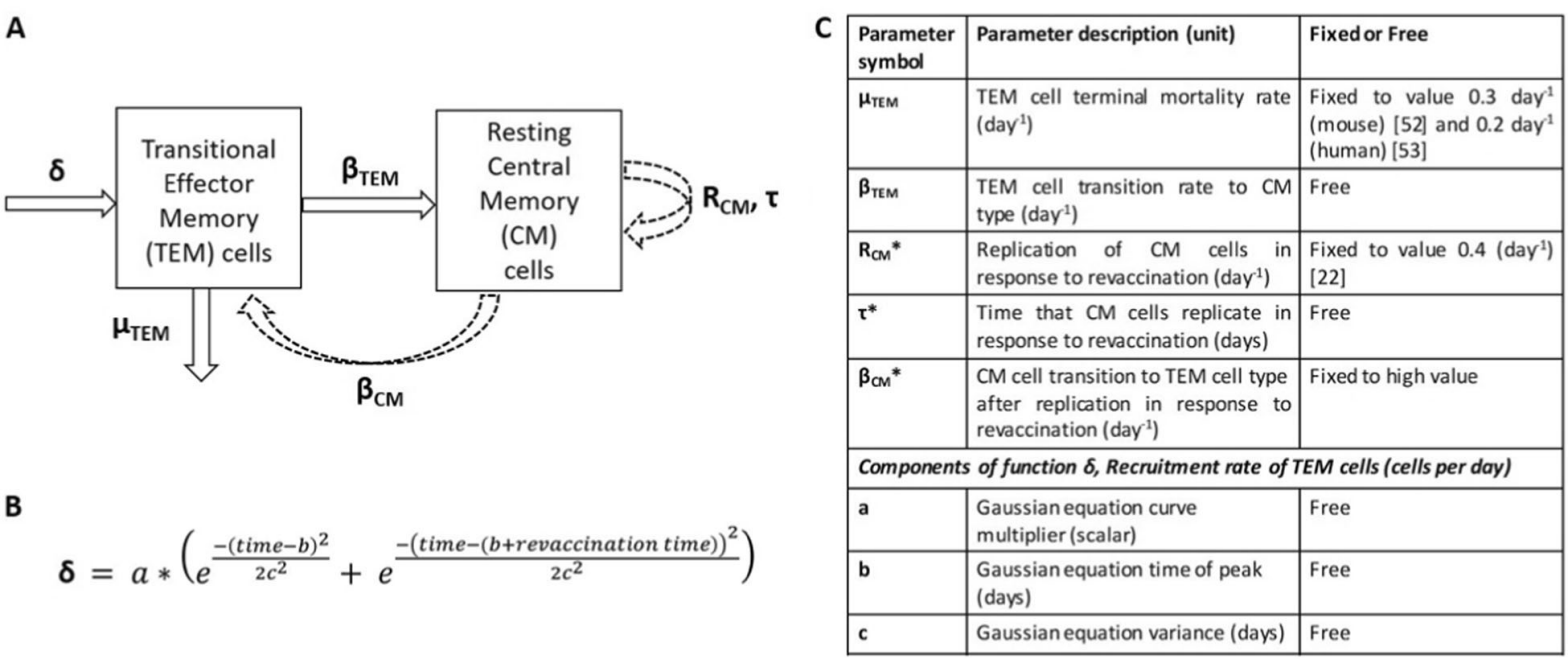
**a** Conceptual schematic of the mechanisms of the immune response dynamics of two IFN-γ secreting CD4+ T cell populations after primary and re-vaccination to be captured by the IS/ID mathematical model. Dashed arrows correspond to T cell dynamics as a result of only revaccination. **b** Gaussian equation describing the TEM cell recruitment parameter *δ*. **c** Table of key model parameters. Model parameters are either fixed to a value from literature (*μ*_TEM_ and *R*_CM_), to an assumed value (*β*_CM_) or free to be estimated using NLMEM (*β*_TEM_, τ, and the parameters that comprise *δ* (**a**, **b**, **c**)). Asterisked parameter symbols correspond to those resulting from only revaccination. The IS/ID model equations can be found in the supplementary material

In anticipation of the release of the dose-ranging clinical trial data (NCT01865487), the aim of this work was to employ a novel IS/ID model to translate H56 + IC31 TB vaccine IFN-γ immune responses from mice to predict the most immunogenic dose in humans. The IS/ID model described the IFN-γ response dynamics of two CD4+ T cell populations induced following vaccination: transitional effector memory (TEM) and resting “central” memory (CM). Briefly, after primary vaccination, cells were recruited into the TEM compartment, where they either transitioned into CM type or entered into a terminal phase. Following revaccination, this process is repeated. Simultaneously, CM cells replicated and entered back to the TEM pool and eventually, the CM pool (see Methods for description of the model, Fig. 1). We fitted our model to IFN-γ data following two vaccinations with TB vaccine H56 adjuvanted with IC31 (H56 + IC31) in mice and humans, and H1 + IC31 data in humans. The model was then used to predict the most immunogenic dose in humans.

Our analysis was in two stages. In analysis 1, the model was fitted to the mouse data stratified by dose group and to the limited dose data on humans. In analysis 2, we used our fitted models to predict the most immunological dose in humans.

## Results

### Analysis 1: Fitting the IS/ID model to the mouse data stratified by dose group and the human data

**Fig. 2:**
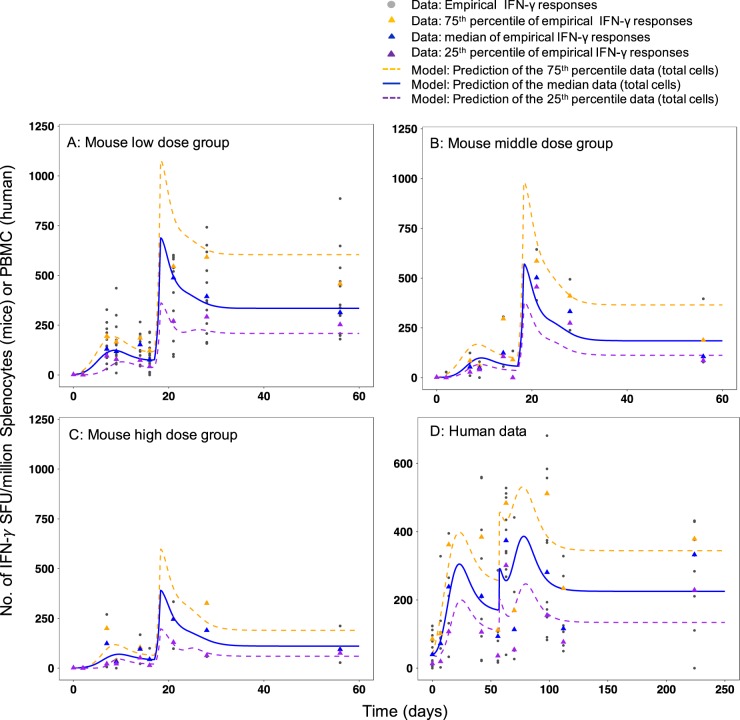
Empirical and model predicted number of IFN-γ secreting CD4+ T cells over time for **a** low dose group (0.1–1 µg H56 + IC31), **b** middle dose group (5 µg H56 + IC31), **c** high dose group (15 µg H56 + IC31) and human dose group (50 µg H56 + IC31). Grey points correspond to number of IFN-γ secreting CD4+ T cells measured over time by ELISPOT assay (in mouse splenocytes A, B and C and PBMC in **d**) after receiving two vaccinations of H56 + IC31 (day 0 and 15, for mice, day 0 and 56 for humans). Median responses over time are marked by a blue triangle, the 75th percentile responses by an orange triangle and the 25th percentile responses by a purple triangle. The model prediction (total cells) fitted to the data in the fitting framework (parameters in Table 1) is plotted against the median data (blue line). The orange and purple dashed lines are the model prediction (total cells) of the 75th and 25th percentiles of the data, a result of the variation in the estimated parameters (standard deviation fixed to 0.5 for all parameters (Table 1))

In Analysis 1, our aim was to fit the IS/ID model (Fig. 1) to the mouse IFN-γ response data stratified by dose group (analysis 1i) and the human data (analysis 1ii) to quantify the IFN-γ response dynamics. For analysis 1i, the best parameter set of the model, was when the TEM to CM cell transition rate (*β*_TEM_) differed by mouse dose group (Fig. 1, Table 1 and Table S1). Figure 2 shows the model predicted IFN-γ response for the low (Fig. 2a), middle (Fig. 2b) and high (Fig. 2c) mouse dose groups (VPC and diagnostic plots in Figures S1–S3). For analysis 1ii parameter estimates for all free parameters (*N* = 5, Fig. 1) were established for the human data (Table 1). Due to the smaller sample size of the human data, parameters that determined the rate of transition of TEM to CM cells (*β*_TEM_) and CM cells replication time (*τ*) were not identifiably estimated (RSE column value > 30%). Figure 2d and the VPC (Figure S4) shows that the model predicted IFN-γ responses from this parameter set (Table 1) was a good description of the median data, despite the wide variability over time of the human responses. See Figures S5–S8 for further diagnostic plots and model predictions for each participant. Model predictions for the 25th and 75th percentiles of the data (Fig. 2a–d) were not as well estimated as the medians for both species because the parameter standard deviations (to account for random effects due to between subject variability (BSV) in response) were fixed at 0.5 throughout. Despite this, the VPC diagnostic plots show the model predictions adequately cover the spread of the data (Fig. 2, S1 and S4). These results imply the model is a good description of the IFN-γ response dynamics for both species and that there is a difference in the model parameters between the mouse dose groups (analysis 1i).

**Table 1:** Population parameters for mice and humans from model fitting (analysis 1)

IS/ID model (Fig. 1) parameter description (unit)	Analysis 1i: Mouse			Analysis1ii: Human	
	Dose group (dose amount)	Parameter value	RSE (%)	Parameter value	RSE (%)
Death rate of transitional effector memory cells, *μ*_TEM_ (per day)		0.3 (NE) (a)	–	0.2 (NE) (b)	–
Transition rate from transitional effector to central memory cell type, *β*_TEM_ (per day)	Low (0.1–1 μg H56 + IC31)	0.23 (E)	30	0.022 (E)	27
	Middle (5 μg H56 + IC31)	0.15 (E)	29		
	High (15 μg H56 + IC31)	0.05 (E)	26		
Replication rate of central memory cells (per day), *R*_CM_		0.4 (NE) (c)	−	0.4 (NE) (c)	−
Central memory cell replication time, *τ* (days)		1.15 (E)	15	0.5 (E)	30
Transition rate from central memory to transitional effector type, *β*_CM_ (per day)		10 (NE)^d^	−	10 (NE)^d^	−
Recruitment of transitional effector rate *δ*: Gaussian equation scalar, a (# cells)		100 (E)	13	58 (E)	23
Recruitment of transitional effector rate *δ*: Gaussian equation mean, b (days)		6.2 (E)	10	20.1 (E)	20
Recruitment of transitional effector rate *δ*: Gaussian equation variance, c (days)		1 (E)	7	9.8 (E)	13

Parameters estimated using the nonlinear mixed effects modelling (NLMEM) framework are indicated with an (E). Those that were not estimated (fixed to a value found in literature or under a model assumption) are indicated with an (NE), their values come from the following sources/assumptions: (a)=ref.^52^, (b)=ref.^53^ and (c)=ref.^22^

*RSE* relative standard error

^d^Fixed to be high, at a value of 10 cells per day. All estimated model parameter standard deviations were fixed at 0.5

### Analysis 2: Use fitted mathematical models in analysis 1, and a vaccine dose allometric scaling assumption, to predict the human immune response dynamics and predict the most immunogenic dose in humans

**Fig. 3:**
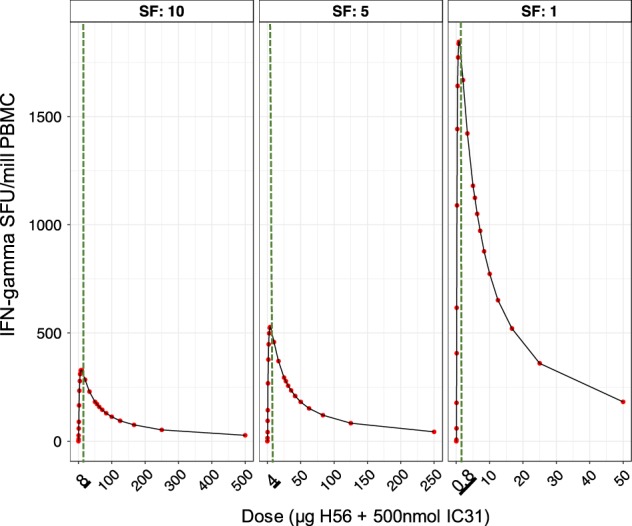
Human predicted H56 + IC31 dose vs. IFN-γ response curve at a late time point (day 224) based on the mouse dose ranging data. Red points are the predicted median number of total IFN-γ secreting CD4+ T cells by the IS/ID model for a range of doses. The green vertical dashed line is the most immunogenic dose in the dose–response curve, the value of which is underlined in the *x*-axis. Each panel shows the results for dose allometric scaling factors of 10, 5 and 1 (for the whole range of scaling factors 1–10, see Figure S10)

Our aim in analysis 2 was to predict human IFN-γ response dynamics for further doses, based on the mouse dose-dependent responses. In analysis 1i, we showed the estimated parameter that determined the rate of transition of TEM to CM cells (*β*_TEM_) was different over the mouse dose groups. In analysis 2, we aimed to establish the population *β*_TEM_ value vs. dose curve (for simplicity, we omit the BSV of *β*_TEM_). In order to fully define this curve, we included *β*_TEM_ for zero H56 + IC31 dose which we assumed was close to zero (with all other model parameters in Table 1) as the IFN-γ response profile for zero dose is flat (Figure S11). We used a peaked curve to describe the population *β*_TEM_ vs. dose curve and predicted further values for *β*_TEM_ for mouse doses ranging 0.01–50 μg H56 + IC31 (see supplementary for model and Figure S9, Table S2). Assuming a dose allometric scaling factor of ten, we calculated the percentage changes in mouse *β*_TEM_ values from the 5 μg to all other doses in the 0.01–50 μg range (Table S2). We applied these percentage changes to the estimated value of *β*_TEM_ for the 50 μg H56/H1 + IC31 human response data (analysis 1ii, Table 1) to predict the human *β*_TEM_ values across a human dose range of 0.1–500 μg H56 + IC31 (Table S2). Using these *β*_TEM_ values in the IS/ID model, we predicted human dose–response curve at a late time point (Fig. 3), which suggested the most immunogenic human dose was 8 μg H56 + IC31 (Fig. 3). In line with the proposed range of dose allometric scaling factor for the H-series, when the scaling factor was varied from 1 to 10, the range of most immunogenic doses was 0.8–8 μg H56 + IC31 (Fig. 3 for scaling factors 1, 5 and 10). These results imply that, based on the mouse dose–response data and accounting for the potential variation of the H56 mouse to human vaccine dose scaling factor, a low dose (between 0.8–8 μg H56 + IC31) in humans may be more immunogenic than higher doses. This should now be verified clinically.

## Discussion

In this work, mathematical models were successfully fitted to animal and human TB vaccine IFN-γ data. Based on the changes in model parameters by mouse H56 + IC31 dose and by varying the dose allometric scaling factor between mouse and humans, we established that, at a late time point (224 days) doses of 0.8–8 μg H56 + IC31 in humans may be the most immunogenic.

Preliminary empirical results from the phase 1/2a clinical dose ranging study of H56 + 500 nmol IC31 (ClinicalTrials.gov no. NCT01865487) may support our model predictions (unpublished, personal communication, Thomas Scriba). These preliminary trial findings from NCT01865487 suggest that doses 5, 15 and 50 μg H56 + IC31 were equally immunogenic in healthy, BCG vaccinated participants, and therefore developers have decided to use 5 μg H56 + IC31 in future clinical trials, rather than 50 μg in previous trials. If these preliminary findings are confirmed, they may support the utility of IS/ID modelling. It must be noted, that these results are preliminary, and empirical samples sizes were small.

A key strength of this work was the application of mathematical modelling techniques to vaccine data that are rarely explored quantitatively. We used established, robust quantitative and statistical frameworks (compartmental mathematical models with NLMEM^18^) to explore and translate the complex biological dynamics between species, giving an early example of the utility of IS/ID modelling. We present here the first example of the allometric mapping between vaccine immune dynamics between mice and humans through the mapping of estimated model parameters between the two species.

Although vaccine IS/ID modelling is analogous to drug PK/PD modelling, there are key differences between the two. For example, we do not use data on how the vaccine distributes in the body (PK), but the stimulation of the immune response as a result of the vaccine exposure (IS) and the resulting response dynamics (ID). The similarity is the use of mathematical models to represent the biological processes and the statistical framework used for model parameter estimation.

We made the following key assumptions in this work. Our model was a highly simplified version of the complexities of the T cell response following vaccination. Our model assumes a linear progression from TEM to CM cell phenotype.^19–21^ However, an alternative model has been suggested, whereby TEM and CM cells are initiated simultaneously after vaccination.^22–24^ These assumptions were necessary to avoid over-parameterisation given the data sample sizes available to us. See supplementary discussion for further model structure assumptions and their impact (Table S3).

Unlike drug dose–response, which is commonly saturating,^10^ we observe a peaked dose–response curve for H56 + IC31 which we show in ref. ^7^ As an explanation of this, Lindenstrøm et al. showed that T cells after high dose of H56 + CAF01 tended more towards an exhaustive state, i.e. reduced functional avidity and increased differentiation into a terminal state.^25^ Our results reflect this, as the rate at which TEM cells (terminal, short-lived) transition to CM cells (long-lived) was lower for higher doses; increasing the amount of terminal cells and minimising the magnitude of response.

There were weaknesses in our work. Small data sample sizes meant we had to firstly, group the mouse dose data in analysis 1, limiting our conclusions on the full range of doses we tested. Secondly, due to the small human dataset (*N* = 18), one of the model parameters was not identifiably estimated, therefore the results of the model fit to the human data should be approached with caution.

There are several areas for future research. The current (antigen) dose allometric scaling factor between mouse and humans for the H-series vaccines is assumed to be 10.^26–28^ A dose allometric scaling factor between mouse and human of 10 has also been used for other vaccines^29–35^ or between 1 and 5,^36–45^ which supports our range of dose allometric scaling factors (1–10). However, to our knowledge no formal assessment of this scaling factor has been undertaken. Our long-term aim is to use IS/ID modelling to predict a likely human dose that can be easily confirmed based only on mouse dose–responses data. However, in the early stages, without extensive allometric knowledge of vaccine responses, this will be an iterative process between modelling and empirical validation before we can achieve this.

We use the frequency of IFN-γ secreting CD4+ T cells measured using the ELISPOT assay as our chosen immune response readout to reflect the current convention in TB vaccine development for dose selection. Although a controversial choice, IFN-γ is a cytokine shown to be associated with control of infection or decreased risk of TB disease.^5,46^ Flow cytometry could provide information on other cytokine types, which could be incorporated into a more complex network model which can provide better understanding of T-cell dynamics. For example, flow cytometry could be conducted to characterise the relative number of complex phenotypic cell types (TEM or CM) over time to further parameterise this model, specifically the transition rate from TEM to CM, *β*_TEM_. Additionally, data on innate cell processes enable us to adapt the immunostimulation parameter (∂) to be biological representative rather than a statistical curve.

In this work, we did not consider different human subpopulations based on geographic location, age, HIV positive status or Latent TB Infection. Additionally, we did not consider an alternative route of administration or change of adjuvant dose. This was due to lack of data and in order to maintain a simple first example of IS/ID modelling. When data are available and provided the same IS/ID model is appropriate, the model can be re-fitted to the data and the further subpopulations treated as population covariates. In general, the IS/ID framework is adaptable to any vaccine data, provided a model can be developed to represent the immune mechanism elicited.

IS/ID modelling could be used to explore the effects of timing of a subsequent vaccination, providing insight into the opportune time to boost vaccine responses, which can then be empirically verified, a common task in MBDD.

In summary, using a mathematical model within a new IS/ID framework, we predicted that low doses of H-series TB vaccine may increase immune response in humans based on animal data. Forthcoming empirical clinical evaluations may support this prediction. We have illustrated that mathematical modelling may be a novel and potentially revolutionary tool to predict most immunogenic vaccine dose, and accelerate vaccine development.

## Methods

### Data

Full details of mouse IFN-γ response data are in ref.^7^ Briefly, female CB6F1 mice were given five doses, 0.1, 0.5, 1, 5, or 15 μg H56 adjuvanted with 100 nmol IC31^®^ (supplied by SSI on behalf of Valneva Austria GmBH; hereafter designated H56 + IC31) plus a control dose of 0 μg H56 + IC31, at day 0 and 15. Data on the number of H56 antigen stimulated IFN-γ secreting CD4+ T cells (in spot forming units (SFU)) per 1 million splenocytes measured by an ex vivo IFN-γ Enzyme-Linked ImmunoSpot (ELISPOT) assay, were taken at eight time points over 56 days (Figure S11 and supplementary methods). Mouse dose groups were: low (0.1, 0.5 and 1 μg H56 + IC31), middle (5 μg H56 + IC31) and high (15 μg H56 + IC31). This plot shows a trend in the longitudinal IFN-γ profile by dose (Figure S11).

Human IFN-γ response data was pooled from phase I clinical trials for the vaccines H56 + IC31 (,^47^ ClinicalTrials.gov no. NCT01967134) (*N* = 8) and H1 + IC31 (^48^ ClinicalTrials.gov no. NCT00929396) (*N* = 10). H1 is comprised of a subset of the H56 antigens.^49^ For both vaccine trials, primary vaccination was administered intramuscularly on day 0 and revaccination, day 56, both at a dose of 50 μg of the vaccine antigen (H1 or H56) and 500 nmol IC31 in healthy, BCG vaccinated participants (hereafter, H56/H1 + IC31). IFN-γ responses were measured using ELISPOT in SFU per 1 million Peripheral Blood Mononuclear Cells (PBMC), taken until day 224 (Figure S12). Further trial information can be found in Table S4.

The adjuvant dose remained constant across antigen dose for both species (100 and 500 nmol IC31 in mice and humans, respectively).

Data collection for mice and humans was conducted in accordance with ethical approval provided by parties outlined in the supplementary methods (data section).

### Immune response mechanism to be represented by the mathematical vaccine IS/ID model

We assumed the mechanisms of the IFN-γ response dynamics were attributed to two CD4+ T cell populations induced following vaccination: TEM^23^ which had effector functionality (activated to produce IFN-γ^24^) and were short-lived and resting CM (Fig. 1). We used an ordinary differential equation model to describe these mechanisms. Conceptually, we assumed following primary vaccination, cells were recruited as transitional cells and entered the TEM cells population (TEM) at rate *δ*. TEM cells then either died, at rate *μ*_TEM_, or transitioned into CM cells at rate *β*_TEM_. CM cells were assumed not to die over the short duration modelled (60 and 250 days in mice and humans, respectively). Following revaccination, transitional cells entering the TEM population were again recruited at rate *δ*, and CM cells replicated at a rate *R*_CM_ for *τ* days. The time that replication occurred for, *τ*, was dependent on the CM population size at time of revaccination. Following replication, CM cells were recruited back to a TEM pool at rate *β*_CM_. As with primary vaccination, TEM cells transition to CM cells at rate *β*_TEM_ following revaccination. As stimulation of T cell responses is delayed following vaccination (due to immune processes such as vaccine antigen trafficking and presentation^50,51^) and does not last indefinitely,^51^ we assumed the TEM cell recruitment rate, *δ*, was nonlinear. *δ* was initiated at time of primary and re-vaccination and was assumed to be the same at both vaccination points.

As CM cells are known to be essentially non-proliferating in the host until stimulated by antigen;^22^ we assumed they contributed to IFN-γ production, because the ELISPOT assay uses the vaccine antigens to stimulate all potentially IFN-γ secreting CD4+ T-cells. To reflect this, the IFN-γ immune response predicted by the mathematical model was assumed equal to the sum of the number of TEM and CM cell populations over time. To account for the potential non-zero baseline responses, the initial TEM cell count was fixed at the median cell count for mice and humans, separately.

The death rate of the TEM cells (*μ*_TEM_) was fixed to values found in literature for mice^52^ and humans,^53^ separately. For both species, the replication rate of the CM cells, *R*_CM_, was fixed to one replicate every 10 h^22^ and the transition rate to TEM pool following replication post revaccination, *β*_CM_, was assumed to be fast at a value of 10 cells per day. All other parameters were free to be estimated. For a conceptual schema of the assumed mechanisms and parameter value description, see Fig. 1.

### Model fitting

The model was fit to the ELISPOT data for both mouse and humans, to quantify the IFN-γ response dynamics. Model fitting was achieved by estimating the values of the free parameters that best describe the IFN-γ response data using nonlinear mixed effects modelling (NLMEM)^18^ and the SAEM algorithm implemented in the software Monolix v. 4.3.3.^54^ SAEM uses maximum likelihood methods to estimate the model parameters that best describe the population typical IFN-γ response and the BSV.^18^ For a technical description of NLMEM see the supplementary methods. The NLMEM statistical model was as follows. A combined residual error model was chosen to describe the random effects due to within subject variability of the responses and no correlations of the random effects were considered necessary in the analysis (data not shown). Model parameters were considered well estimated if their relative standard error (RSE) was <30%.^55^ The standard deviations of the model parameters that describe the random effects due to BSV, were not estimated (fixed) at 0.5 unless there was power to do so once the population typical parameter estimates were well estimated (see supplementary methods for further description).

Model selection was carried out using Bayesian Information Criteria (BIC) value assessment, where a lower BIC value was indicative of a better fit. Evaluation of the model’s ability to describe the data was assessed primarily using the visual predictive check (VPC) and further diagnostic plots (see supplementary methods for description).

Firstly, to determine a form for the recruitment of TEM cells (parameter *δ*, Fig. 1) we tested two nonlinear equations in the model fitted to the pooled mouse data; a Gaussian equation and a gamma probability density function (PDF) equation. We also tested the replacement of rate *δ* with a naïve T cell compartment, whereby naïve cells replicate for *τ*_N_ days before transitioning to TEM at rate *β*_N_ (for mathematical description of the forms, see supplementary). All parameters within the forms of *δ* were to be estimated. When the *δ* forms were fitted to the mouse data (for primary and revaccination), the lowest BIC value was with the Gaussian equation (Fig. 1, Table S5):

$$a \ast \left( {e^{\frac{{ - \left( {t - b} \right)^2}}{{2c^2}}} + e^{\frac{{ - \left( {t - \left( {b + t_{\mathrm {r}}} \right)} \right)^2}}{{2c^2}}}} \right)$$

where *a* is a scalar, *b*, the Gaussian equation mean, *c*, the variance, *t* is time, measured in days and *t*_r_ is revaccination time measured in days. When this form was used on the pooled mouse data, the model predicted the data well (Table S6, Figures S13–S14). We also conducted a likelihood sensitivity analysis of the model parameters on the pooled mouse data (Figure S15 and Table S7).

### Model code availability

The Monolix code (MLXTRAN code) used to fit the IS/ID model to the data is available in the supplementary methods.

### Analyses

#### Analysis 1: Fitting the IS/ID model to the mouse data stratified by dose group and the human data

In analysis 1, we aimed to fit the IS/ID model to the mouse data stratified by dose group (analysis 1i) and the human data (analysis 1ii). We used the likelihood ratio test (LRT) compared to the pooled mouse fit (see supplementary) to identify which model parameters should be stratified by dose group. The human data set was pooled across vaccine H1 + IC31 and H56 + IC31, as the two vaccines are known to have a similar immunological profile^47^ (see Table S8 for analysis on the human data stratified by vaccine type to validate this assumption).

#### Analysis 2: Use fitted mathematical models in analysis 1, and a vaccine dose allometric scaling assumption, to predict the human immune response dynamics and predict the most immunogenic dose in humans

In analysis 2, the estimated model parameters identified for the dose groups in mice and for the one dose in humans (analysis 1) were used to predict the IFN-γ response in humans for a range of doses. We followed the steps:

1. We used a statistical model to represent the change in the mouse dose-dependent population parameter(s) (DDPP(s) for ease) values by dose (estimated in analysis 1i). We then extrapolated further DDPP(s) values for doses in a range of 0.01–50 μg of H56 + IC31. For simplicity, BSV of the DDPP(s) will be excluded in this analysis.
2. As the current (antigen) dose allometric scaling factor between mouse and humans for the H-series vaccines is assumed to be 10,^26–28^ we calculated that the human dose range, based on the mouse dose range in step 1 (0.01–50 μg H56 + IC31) and this scaling factor, was 0.1–500 μg H56 + IC31.
3. As we assumed a scaling factor of 10, the 50 μg H56/H1 + IC31 dose given to humans was equivalent to the 5 μg H56 + IC31 dose group in the mice. We calculated the percentage change between the mouse DDPP(s) values for the 5 μg H56 + IC31 dose and the mouse DDPP(s) values for remaining doses between 0.01 and 50 μg H56 + IC31 (found in step 1).
4. To translate the changes in mouse DDPP(s) to the human dose range, we applied the percentage changes found in step 3 to the corresponding human DDPP(s) found in analysis 1ii (for the 50 μg H56/H1 + IC31 dose).
5. Finally, to establish the long-term human dose–response curve and ‘most immunogenic’ human dose we applied the human DDPP(s) found in step 4 into the IS/ID model to predict the IFN-g responses.

To further this analysis and as the dose allometric scaling factor for the H-series could potentially be between 1 and 10 (personal communication, T Evans), we repeated steps 2–5 assuming this range of scaling factor.

### Data availability

The mouse and human data used in this paper is publicly available through publication (ref.^7^ for mouse and refs.^47,48^ for human). Figures of the raw data used in the analysis are in Figures S11 (mouse) and S12 (human).

## Electronic supplementary material

Supplementary material

## Electronic supplementary material

**Supplementary information** accompanies the paper on the *npj Vaccines* website (10.1038/s41541-018-0075-3).
