# Supplementary material for: Using vaccine Immunostimulation/Immunodynamic modelling methods to inform vaccine dose decision-making
Source: NPJ Vaccines. 2018 Sep 17;3:36. doi: 10.1038/s41541-018-0075-3 (PMC6141590; doi:10.1038/s41541-018-0075-3)
Supplement: Supplementary file 1 — Supplementary material [file 41541_2018_75_MOESM1_ESM.pdf]

Supplementary material for: Using vaccine Immunostimulation/Immunodynamic modelling methods to inform vaccine dose decision-making

Additional Results

Analysis 1: Fitting the IS/ID model to the mouse data stratified by dose group and the human data

As described, the LRT was used to establish the mouse covariate model in analysis 1i. The selected model for analysis 1i was the one which satisfied the LRT against the pooled model (see supplementary methods for the model fit to the pooled mouse data, table S6), had all estimated model parameters RSE <30%, and had the lowest -2LL. Table S1 shows the result of indexing dose group on the estimated model parameters from the pooled model (model parameter standard deviations were all 0.5).

-2LL value for pooled model	Dose group indexed on parameter(s)	Results		Difference in -2LL from pooled model (pooled-dose group)	0.01 level significant? (Chi^2 test<br>3 d.f.: crit val = 11.34,<br>6 d.f.: crit val = 16.81,<br>9 d.f.: crit val = 21.67,<br>12 d.f.: crit val = 26.22,<br>15 d.f.: crit val = 30.58)
		Parameters with RSE >30%	-2LL		
2344	a	None	2322	22	(3 d.f.) Yes
	b	None	2333	11	(3 d.f.) No
	c	None	2322	22	(3 d.f.) Yes
	β_{TEM}	None	2320	24	(3 d.f.) Yes
	τ	None	2335	9	(3 d.f.) No
	a, b	a	2314	30	(6 d.f.) Yes
	a, c	a	2323	21	(6 d.f.) Yes
	a, β_{TEM}	c	2318	26	(6 d.f.) Yes
	a, τ	None	2322	22	(6 d.f.) Yes
	b, c	None	2325	19	(6 d.f.) Yes
	b, β_{TEM}	None	2323	21	(6 d.f.) Yes
	b, τ	None	2329	15	(6 d.f.) No
	c, β_{TEM}	c	2320	24	(6 d.f.) Yes

	c, τ	c	2320	24	(6 d.f.) Yes
	β_{TEM}, τ	c	2316	28	(6 d.f.) Yes
	a, b, c	a, b, c, b_{TEM}	2313	31	(9 d.f.) Yes
	a, b, β_{TEM}	a, b_{TEM}	2312	32	(9 d.f.) Yes
	a, b, τ	a, b, τ	2316	28	(9 d.f.) Yes
	a, c, β_{TEM}	a, c, b_{TEM}	2322	22	(9 d.f.) No
	a, c, τ	a, c, t	2325	19	(9 d.f.) No
	a, β_{TEM}, τ	a, c, b_{TEM}	2319	25	(9 d.f.) Yes
	b, c, β_{TEM}	b_{TEM}	2317	27	(9 d.f.) Yes
	b, c, τ	b, c	2316	28	(9 d.f.) Yes
	b, β_{TEM}, τ	β_{TEM}	2312	32	(9 d.f.) Yes
	c, β_{TEM}, τ	b_{TEM}	2322	32	(9 d.f.) No
	a, b, c, β_{TEM}	a, b, c, b_{TEM}	2317	27	(12 d.f.) No
	a, b, c, τ	a, b	2317	27	(12 d.f.) No
	a, b, β_{TEM}, τ	a, b_{TEM}	2315	29	(12 d.f.) No
	a, c, β_{TEM}, τ	a, c, b_{TEM}	2321	23	(12 d.f.) No
	b, c, β_{TEM}, τ	All	2310	34	(12 d.f.) Yes
	a, b, c, β_{TEM}, τ	All	2320	24	(15 d.f.) No

Table S1. Results of indexing the dose group covariate on all combinations of estimated parameters in the mouse pooled model (see table S6)

Table S1 shows that the best covariate model is when dose group was indexed on model parameter β_{TEM} with all model parameter standard deviations fixed to 0.5 (highlighted)(allowing the standard deviations to be estimated led to RSE of one or more parameters >30%).

Diagnostic Plots

The VPC plot, prediction distribution and observed versus predicted response plots can be found in Figures S1-S3.

The VPC shows that for each dosing group (low, middle and high), the model predicts the data well (Figure S1), although with less data per group the VPC is not as well defined. This is due to the small sample size for the high and middle dose groups, as the VPC plot does not summarise all responses, either observed data (green line) and model simulations (blue and orange regions) for all times points which is why the green line, blue and orange regions do not reflect the expected shape of the model prediction, i.e. there is no clear peak after

primary and revaccination as would be expected from the design of the IS/ID model. This is not a reflection of an unidentifiable model fitting, but an artefact of the default settings for the VPC plot in Monolix, where model predictions for small sample sizes are misrepresented. Figure 2A-C is a better depiction of the model prediction versus the observed data. The observed versus predicted response plots in Figure S3 suggest that the model predictions fall in line with the observed data for the dose groups on a population and individual level.

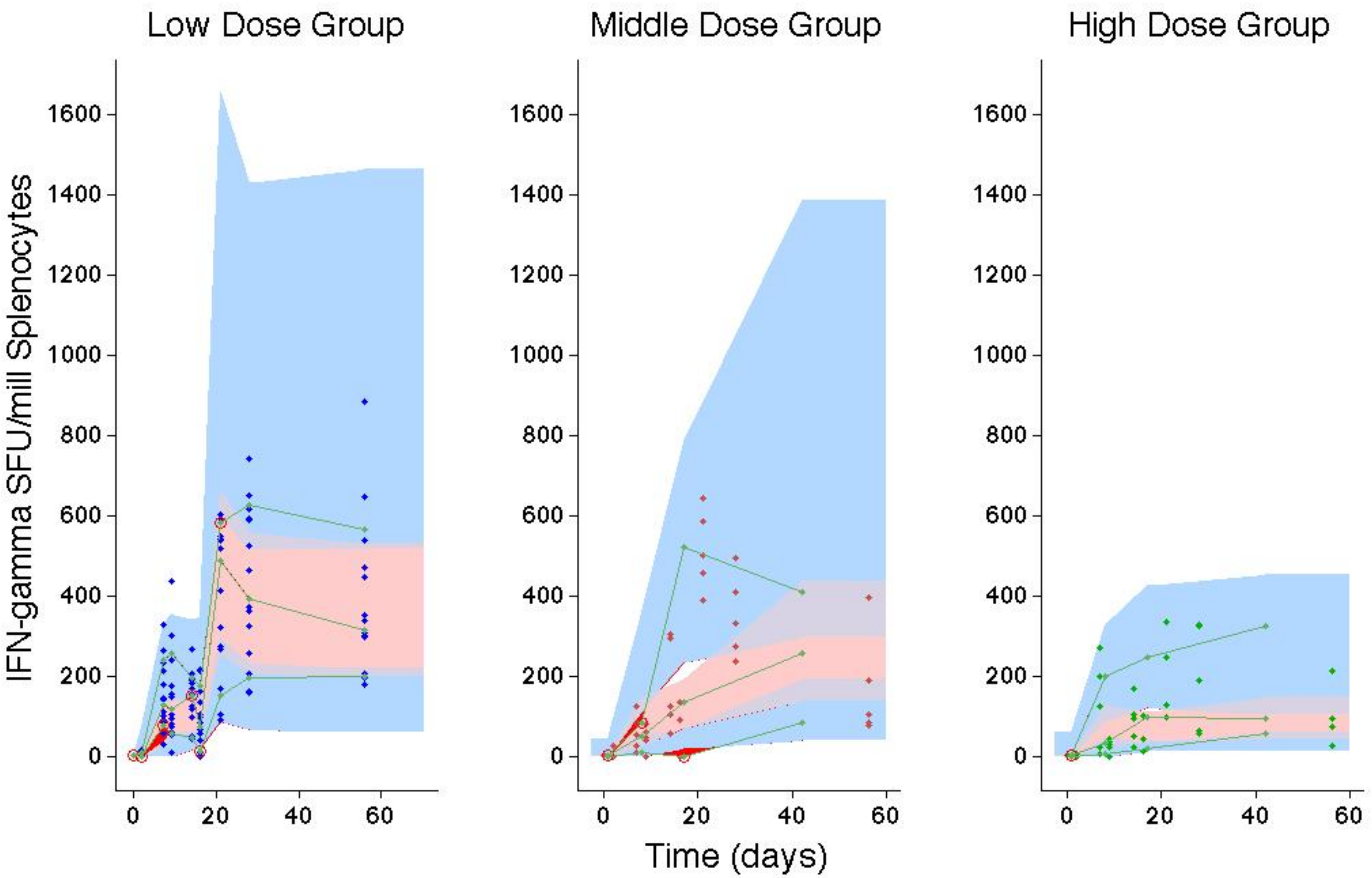

Figure S1. Visual Predictive Check (VPC) plot for the covariate mouse model (dose group indexed on parameter β_{TEM}, see Table S1, estimated parameters in Table 1). Blue points represent the observed data. Blue regions represent the ranges of the 75th and 25th percentiles of the simulated populations. The pink region represents the range of the 50th percentile. The green line links the observed percentiles (25th, 50th and 75th) for each time point. Red regions represent where the observed data falls outside the ranges of the simulated percentiles

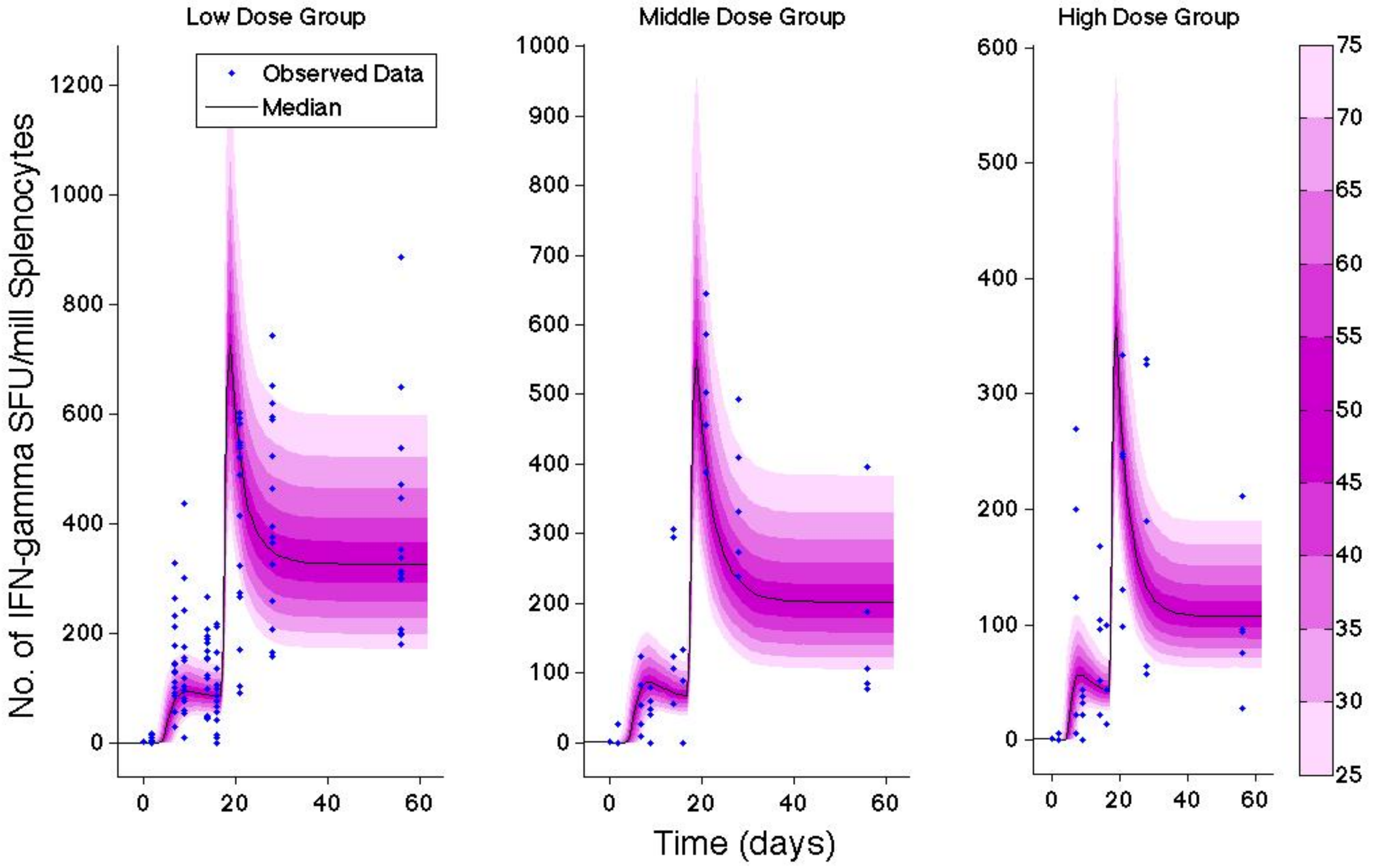

Figure S2. Prediction distribution plot for the fit to the mouse data stratified by dosing group (dose group indexed on parameter β_{TEM}, see Table S1, estimated parameters in Table 1). The blue points represent the data. The bands represent the 25th to 75th percentiles of the theoretical predictions using the estimated population parameters and associated variation for analysis 1i (Table 1). The black line shows the median total cell response prediction. Note, Y-axis not on the same scale.

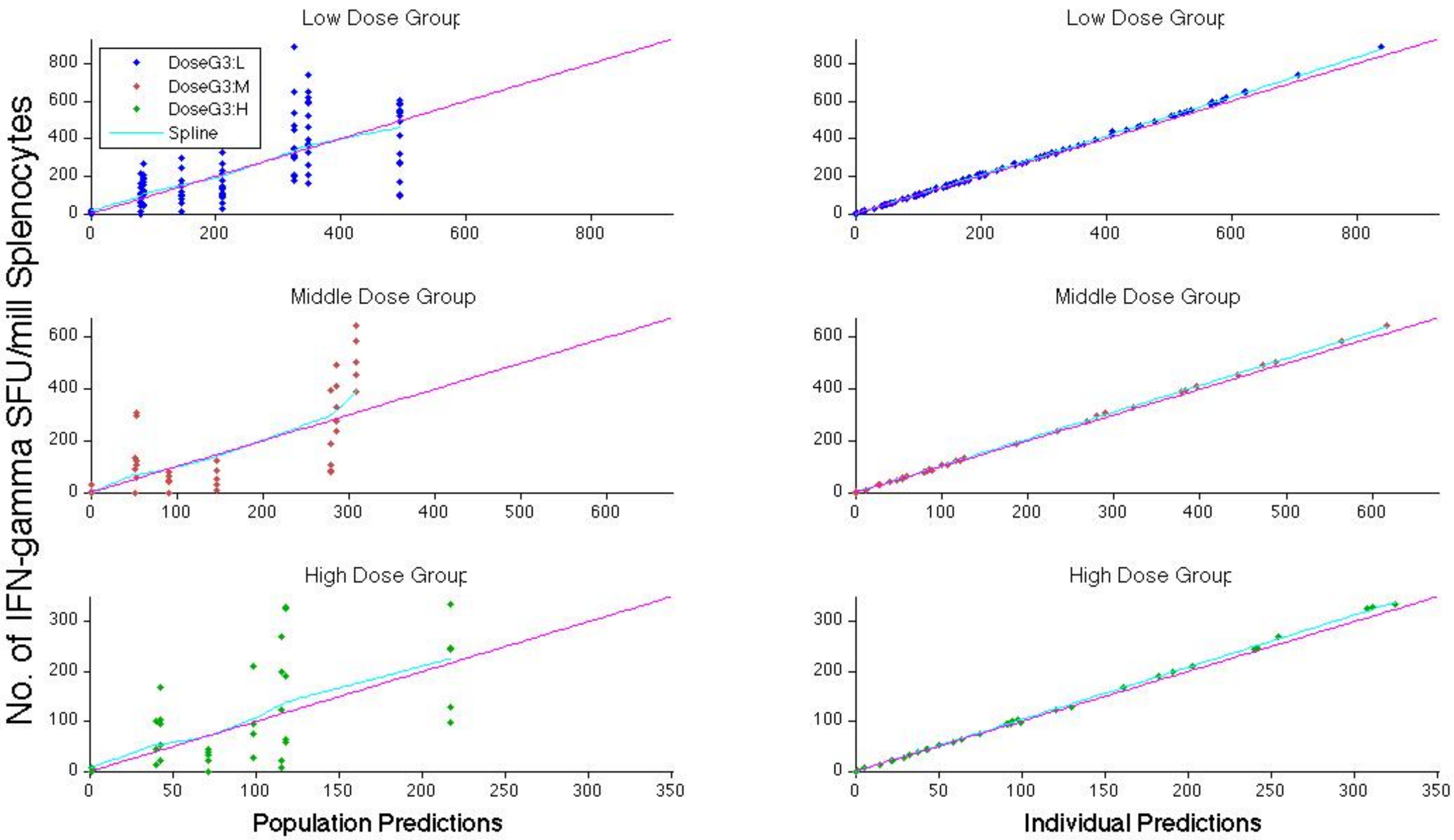

Figure S3. Mouse observed data versus model predicted IFN-γ responses stratified by dose group (dose group indexed on parameter β_{TEM}, see Table S1, estimated parameters in Table 1).

The results of the model fit to the human data can be found in Table 1. As one of the parameters RSE =30% the effects of estimating model parameter standard deviations (to account for BSV) was not tested as it was clear there was not enough data to estimate further parameters.

Diagnostic Plots

The VPC plot, model prediction distribution plot and the observed versus predicted (for the population and individual participants) for the pooled human model can be found in Figures S4-S6.

The VPC plot shows that the simulated model cover the data well except for an underestimation of the median response at the latest time point (Figure S4). This may be due to the fact that there is less data at the latest time point (these are only the H1 responses, not the pooled H56 and H1 responses). Again, due to the small sample size, this VPC plot does not summarise all responses, either observed data (green line) and model simulations (blue and orange regions) for all times points which is why the green line, blue and orange regions do not reflect the shape of the model prediction in Figure 2D of the main paper. Similarly, this is not a reflection of the fitting of the model, but an artefact of the default settings for the VPC plot in Monolix, where model predictions for small sample sizes are misrepresented. However, the expected profile from the IS/ID model can be seen better in the model prediction distribution plot, which suggest the percentiles of the data are adequately covered (Figure S5) despite widely variable responses over time in the human data set. Figure 2D in the main paper shows how the model predictions follow the trend of this variable data. However, similar to the mouse pooled model, as all parameter standard deviations are fixed at 0.5, this may be underestimating the responses in some cases (although the observed versus predicted individual responses suggests the model is a good fit (Figure S6)). The individual plots for each human participant can be found in Figure S7 and S8.

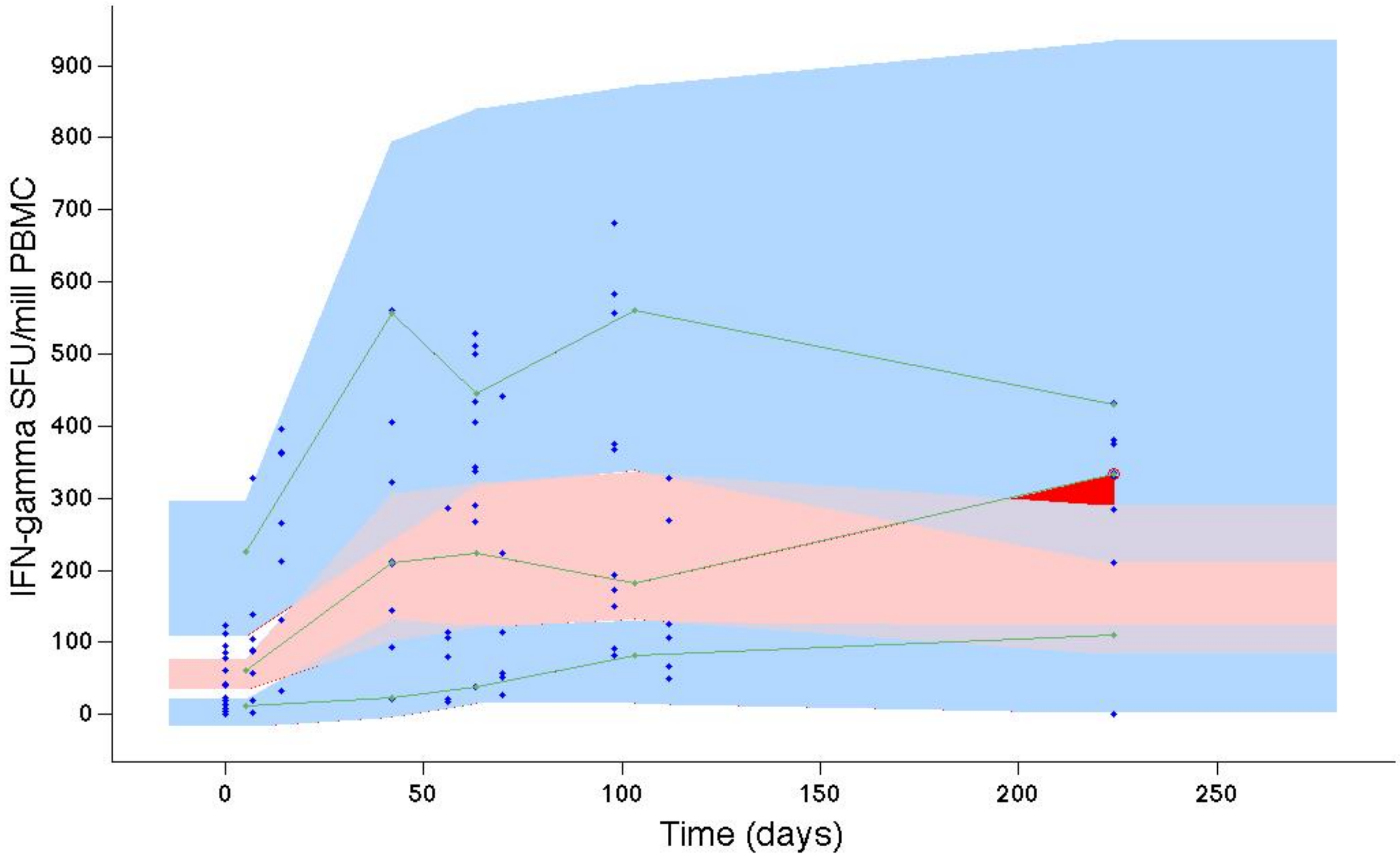

Figure S4. Visual Predictive Check (VPC) plot for the pooled human model (model parameters Table 1). Points represent the observed data. Blue regions represent the ranges of the 75th and 25th percentiles of the simulated populations. The pink region represents the range of the 50th percentile. The green line links the observed percentiles (25th, 50th and 75th) for each time point. Red regions represent where the observed data falls outside the ranges of the simulated percentiles.

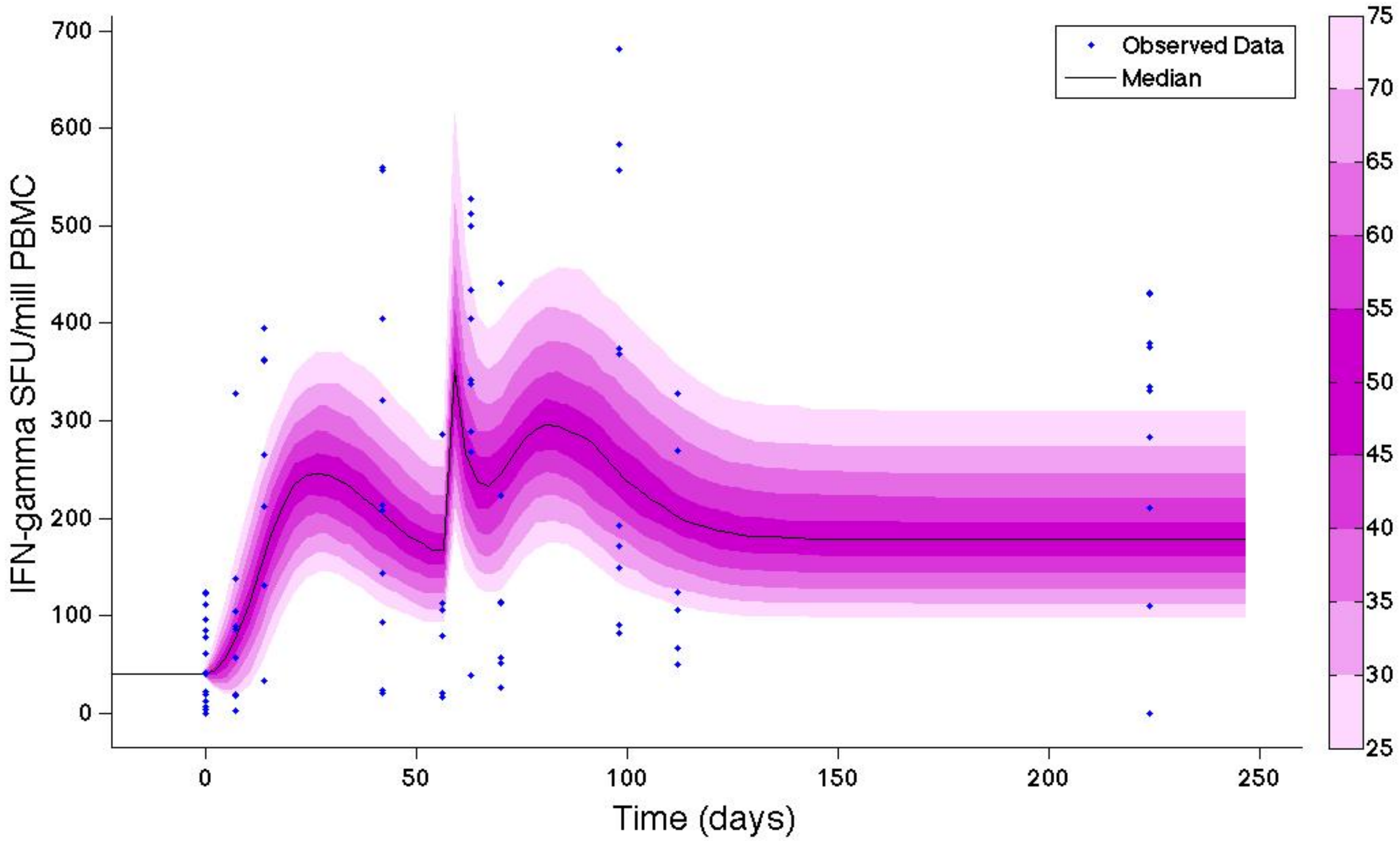

Figure S5. Prediction distribution plot for the fit to the human data. The black points represent the data. The bands represent the 25th to 75th percentiles of the theoretical predictions using the estimated population parameters and associated variation for analysis 2 (Table 1). The black line shows the median total cell response prediction

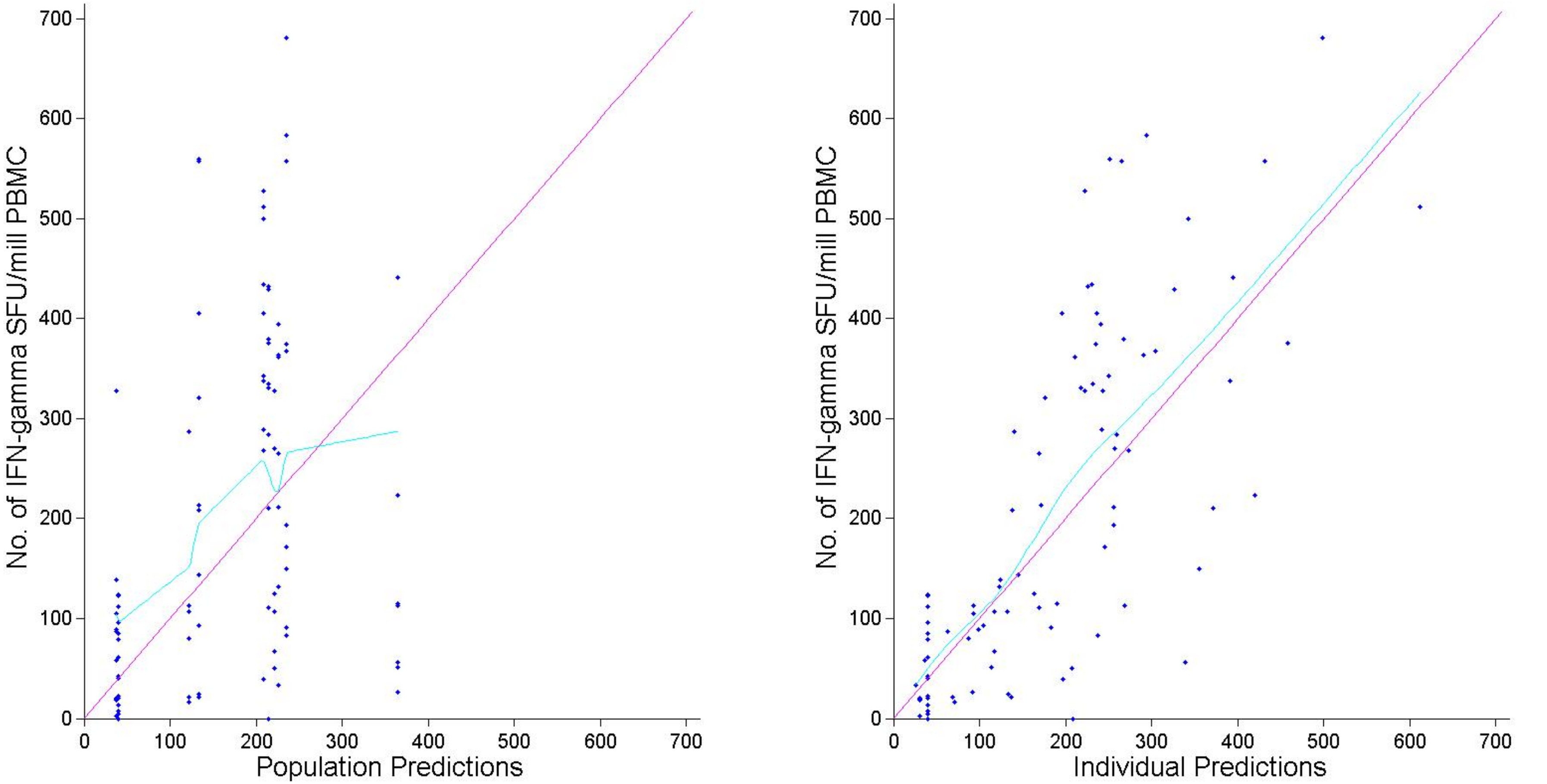

Figure S6. Human observed data versus model predicted IFN-γ responses

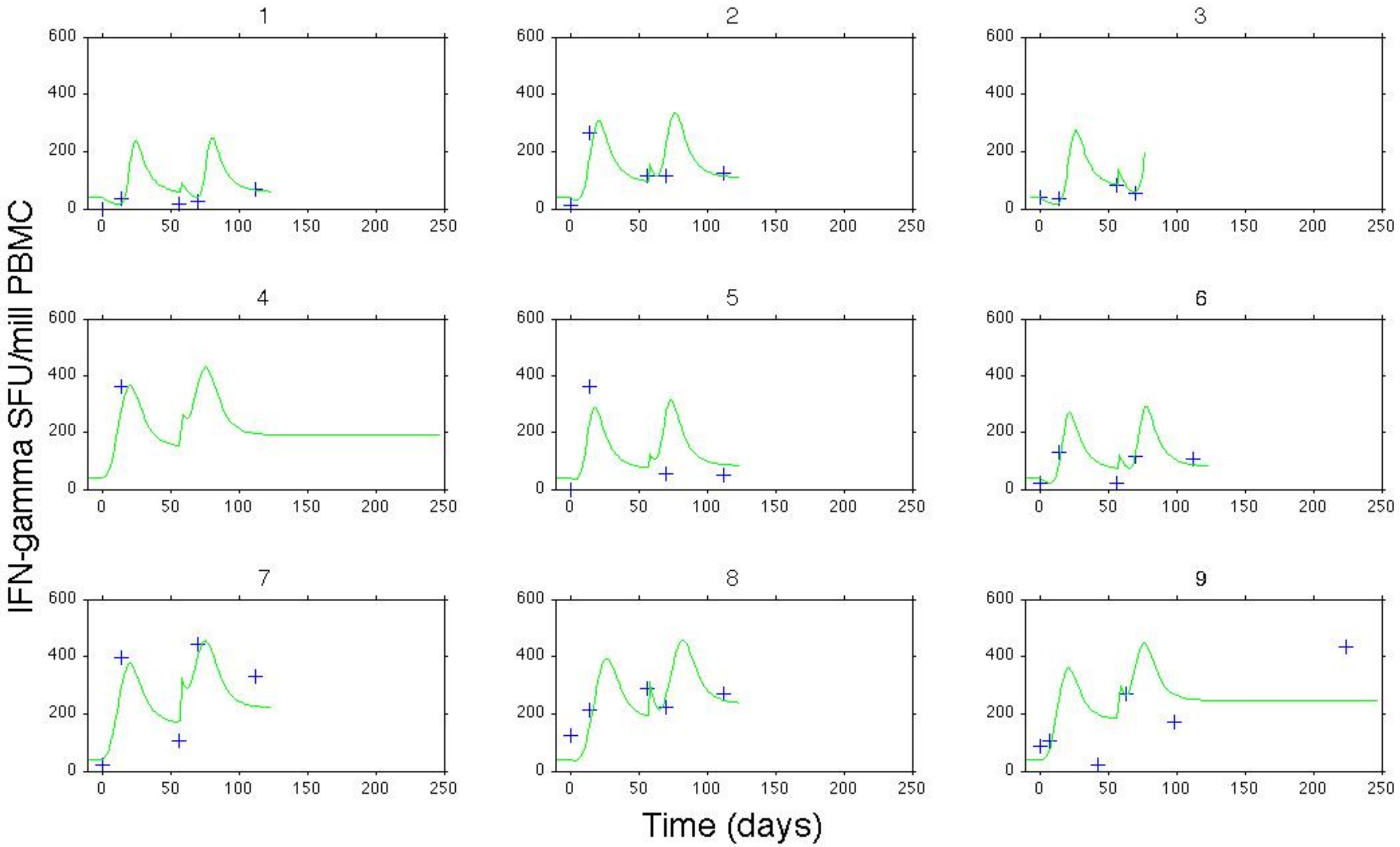

Figure S7. Model predictions for each participant of the human data set. Plot 1 of 2.

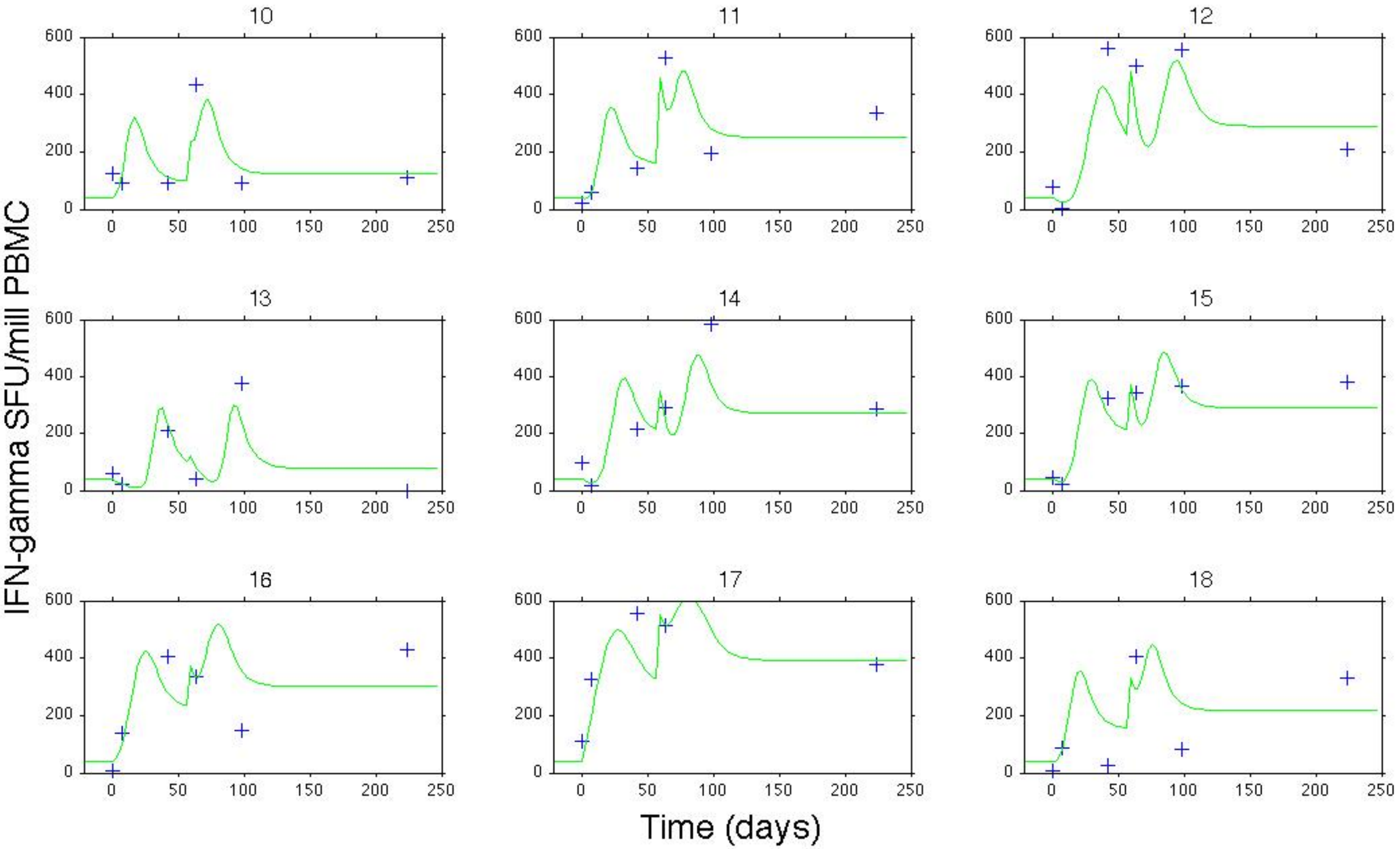

Figure S8. Model predictions for each participant of the human data set. Plot 2 of 2.

Analysis 2: Use fitted mathematical models in analysis 1, and a vaccine dose allometric scaling assumption, to predict the human immune response dynamics and predict the most immunogenic dose in humans

In analysis 2, the estimated model parameters identified for the dose groups in mice and for the one dose in humans (analysis 1) were used to predict the IFN-γ response in humans for a range of doses. The steps to achieve this are outlined in the methods.

We found in analysis 1i, that the dose-dependent parameter was β_{TEM} (table 1). Here we demonstrate how the steps described in the methods were applied to parameter β_{TEM} to predict the human β_{TEM} vs dose and thus the dose response curve for a dose allometric scaling factor of 10 (the steps from the methods section are repeated here in italics):

1. *We used a statistical model to represent the change in the mouse dose-dependent population parameter(s) (DDPP(s) for ease) values (estimated in analysis 1i) by dose. We then extrapolated further DDPP(s) values for doses in a range of 0.01-50 µg of H56+IC31. For simplicity, BSV of the DDPP(s) will be excluded in this analysis.*

   To achieve this step, we estimated three values for β_{TEM} from the mouse data stratified by dose (for the low, middle and high dose group) by fitting the IS/ID model to the empirical mouse data using NLMEM (analysis 1i, Table 1). For step one we assume the average dose value for the low dose group (average of 0.1, 0.5, 1). We also included the β_{TEM} value for the control mouse data (mice who received no H56) which we assumed was low as the IFN-γ response for the zero group was flat (Figure S11). We verified this assumption by fitting the model to the zero dose data keeping all parameters except β_{TEM} fixed to the estimated population β_{TEM} values in Table 1 (with the BSV fixed at 0.5). A low value, approximately 0.004 cells per day was estimated (data not shown). To find a representative equation to the β_{TEM} vs dose curve, we firstly, transformed the dose values using the log transformation: Dose_Trans=(Log10(Dose))+2 (Figure S9A, black points), then using the nls package in R, we fit a gamma pdf equation:

$$\beta_{TEM}\ \ value = S * \frac{rate^{shape}}{\Gamma(shape)} * Dose\_Trans^{(shape-1)} * e^{-rate*Dose\_Trans}$$

Where S is a scalar of the curve and the rate and shape are the parameters of the gamma pdf (figure S9A, solid black line). Using the fitted gamma pdf curve, we were able to predict further values of β_{TEM} for a range of doses (Figure S9A, red points). We then untransformed dose_trans to give a dose range of 0.01-50 ug H56 (Figure S9B, table S2).

For the remaining steps, see Table S2.

2. *As the current (antigen) dose allometric scaling factor between mouse and humans for the H-series vaccines is assumed to be ten [1-3], we calculated that the human dose range, based on the mouse dose range in step 1 (0.01-50 µg H56+IC31) and this scaling factor, was 0.1-500 µg H56+IC31.*
3. *As we assumed a scaling factor of ten, the 50 µg H56/H1+IC31 dose given to humans was equivalent to the 5 µg H56+IC31 dose group in the mice. We calculated the percentage change between the mouse DDPP(s) values for the 5 µg H56+IC31 dose and the DDPP(s) values for remaining doses between 0.01-50 µg H56+IC31 (found in step 1).*
4. *To translate the changes in mouse DDPP(s) found in step 1 to the human dose range, we applied the percentage changes found in step 3 to the corresponding human DDPP(s) found in analysis 1ii (for the 50 µg H56/H1+IC31 dose).*
5. *Finally, to establish the long term human dose response curve and 'most immunogenic' human dose we applied the human DDPP(s) found in step 4 into the IS/ID model to predict the IFN-γ responses.*

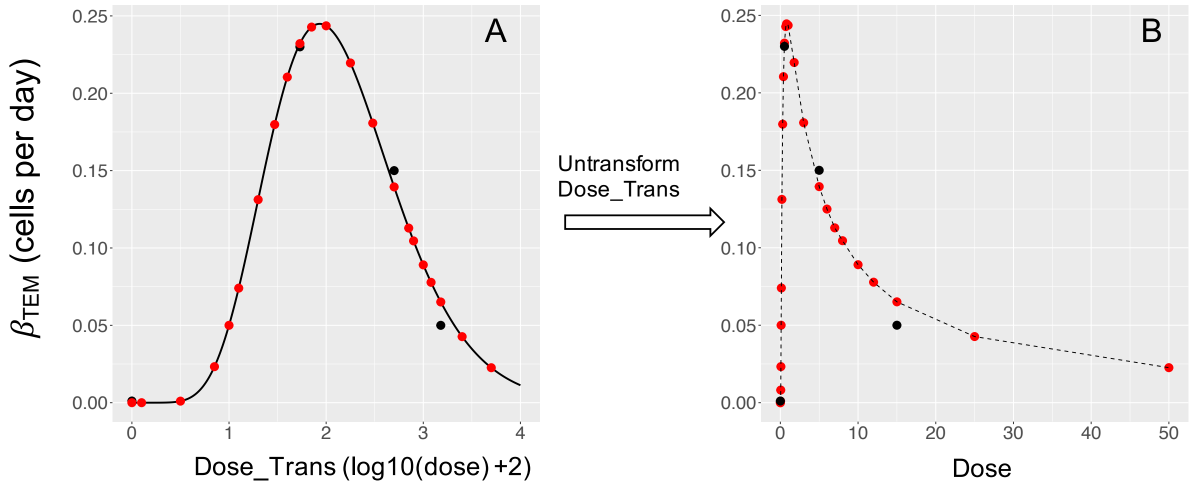

Figure S9. Model for predicting Btem versus Dose_trans (A) and predicted Btem versus Dose (B). Black points are the Btem values found in analysis 1i for the mouse dose groups (Table 1). Black solid line in A is the gamma pdf equation fit to the estimated Btem values from analysis 1i (black points) versus Dose_Trans. Red points are predicted values of Btem for further doses using the gamma PDF equation fit. Black dashed line in B connects the Btem values for the untransformed dose values for ease of view.

**Step 1**		**Step 2**		**Step 3**		**Step 4**	**Step 5**
All predicted mouse doses (untransformed) and predicted btem value at dose (Figure S9B)		Scaling Factor (SF) of mouse dose to human 50micg dose	Predicted human dose using SF*predicted mouse dose	Equivalent dose in mice to human 50 ug dose using 50/SF (btem value at this dose)	% change in btem from the "equivalent" to all other doses in mice	BTEM value at this human dose (% change*btem for human 50 ug)	Human IFN-γ value at day 224 at this BTEM
Dose	Btem						
0	0	10	0	5 (0.139)	-100%	0	0
0.01	0		0.1		-100%	0	0
0.03	0.001		0.3		-99%	0	1
0.05	0.008		0.5		-94%	0.001	10
0.07	0.023		0.7		-83%	0.004	28
0.1	0.05		1		-64%	0.008	61
0.13	0.074		1.3		-47%	0.012	92
0.2	0.131		2		-6%	0.021	170
0.3	0.18		3		29%	0.028	240
0.4	0.21		4		51%	0.033	284
0.53	0.232		5.3		66%	0.037	316
0.7	0.243		7		74%	0.038	332
0.8	0.245		8		75%	0.039	335
1	0.244		10		75%	0.038	333

1.78	0.22		17.8		57%	0.035	298
3	0.181		30		30%	0.029	241
5	0.139		50		0%	0.022	182
5.56	0.125		55.6		-10%	0.02	162
7.14	0.113		71.4		-19%	0.018	145
8.33	0.105		83.3		-25%	0.016	134
10	0.089		100		-36%	0.014	112
12.5	0.078		125		-44%	0.012	97
16.67	0.065		166.7		-53%	0.01	81
25	0.043		250		-69%	0.007	52
50	0.023		500		-84%	0.004	27

Table S2. Steps 1-5 for predicting the human dose response curve at day 224 for a dose allometric scaling factor of 10.

For the remaining scaling factors, which for the H-series could potentially be between one and ten (personal communication, T Evans), we chose doses that would produce approximate values from 1 to 10. For example, for a scaling factor of 9, the dose 50/9=5.556 was predicted in step 1. For the remaining scaling factors (1 to ten), steps 2-5 were repeated. These calculations are not included here.

The human predicted dose response curves are in Figure 3 for Scaling factor 1, 5 and 10. For the dose response curves for the remaining scaling factors, see Figure S10.

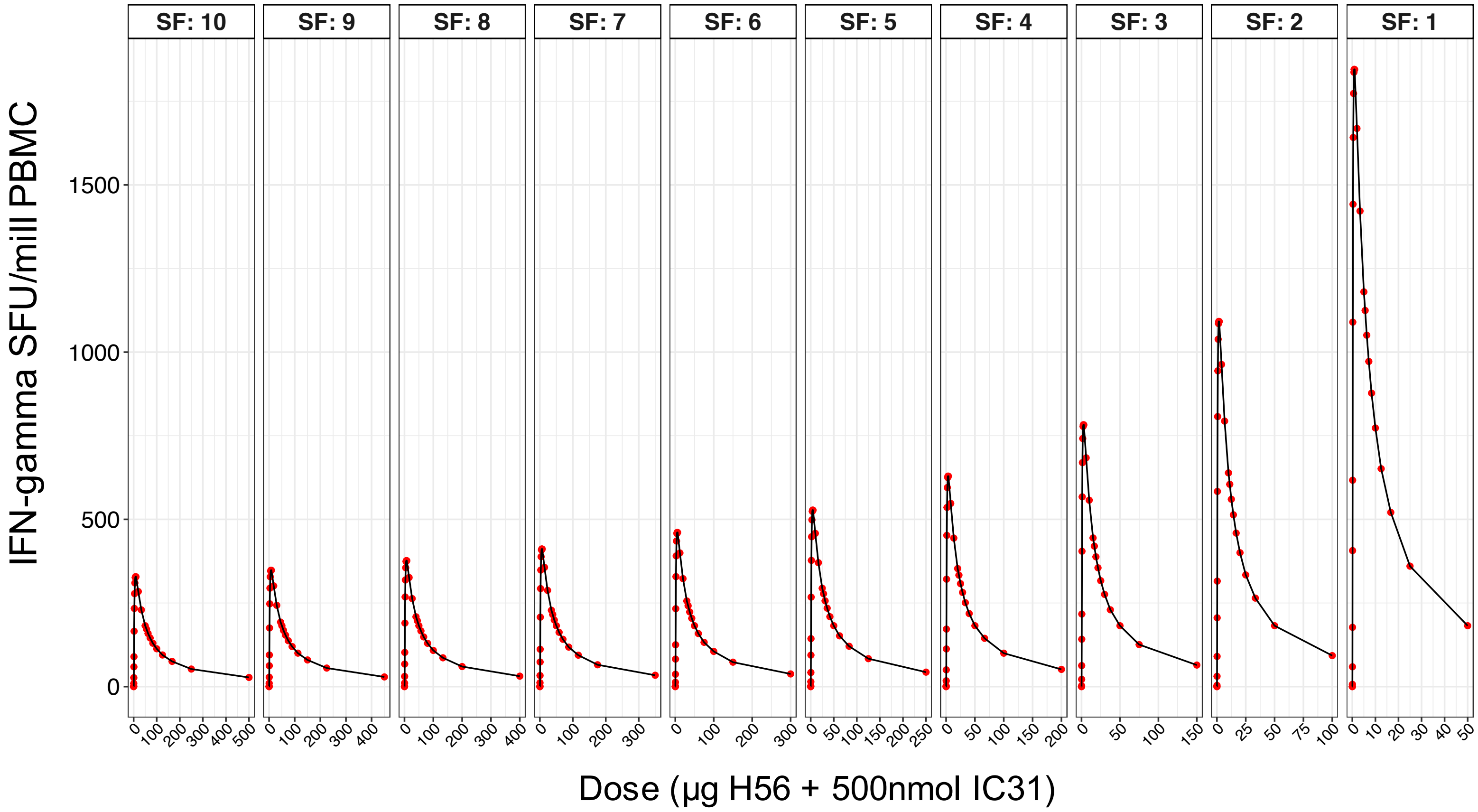

Figure S10. Human predicted H56 dose versus IFN-γ response curve at a late time point (day 224) based on the mouse dose ranging data. Red points are the predicted median no. of total IFN-γ secreting CD4+ T cells by the IS/ID model for a range of doses. The green vertical dashed line is the most immunogenic dose in the dose response curve, the value of which is underlined in the x-axis. Each panel shows the results for dose allometric scaling factors of 10 to 1.

Additional Discussion

Model Assumptions

Key model assumptions from the IS/ID model are outlined in Table S3.

Assumption	Implications for model
**<u>Baseline responses were fixed at the median value</u>**<br>In this model, the initial values for the Transitional Effector Memory cells (TEM_0) were not estimated. This is due to the fact that all mice IFN-γ responses at baseline were based on measurements from one unvaccinated mouse and therefore were all zero. As all human participants in the clinical trials were previously BCG vaccinated and no other human covariates were considered that could impact on a baseline response, the baseline responses were fixed to the median value. This also aided in avoiding over parameterisation compared to the small sample size of the human data.	
**<u>Central Memory (CM) cells do not die</u>**<br>The central memory cell population is assumed to be maintained be a constant turnover, so we assumed the death rate could be omitted from the both the human and mouse model [4]. Although there is evidence to suggest CD4+ long-term memory cells turnover may diminish with time [5,6], we assumed this does not affect the time frame of the model.	Introducing a death rate of memory cells would result in a decline of the long-term responses.

**<u>Replication followed by transition of CM cells after revaccination and rate of transition, β_{CM}</u>**<br>In the model, after revaccination, the CM cells replicated at a fixed rate for time τ, which was estimated in the model fitting stage. Only after replication had occurred, the cells transitioned back to TEM cell type at a rate β_{CM}, which was assumed to be fast. Although this may be a simplification of the host immune response dynamics, it was necessary to assume as we did not have information on β_{CM}. We therefore considered the transition of CM cells to TEM cells as a result of revaccination to be a proliferation followed by a "burst" as opposed to a slower gradual transition (where proliferation and transition occur simultaneously). We believe this assumption is justified as the purpose of CM cells are to mount an immune (in our case, IFN-γ) response faster than a primary response as a result of re-exposure to the antigen (revaccination) [7] and a "burst" response is an effective method to represent this dynamic.	
**<u>IFN-γ responses are not scaled to host body size</u>**<br>The ELISPOT assay readout is conventionally measured per million cells in all species and we considered the model to represent a systemic response regardless of host blood volume, it was not necessary to scale the ELISPOT readout to reflect body size. As our focus was on translating the *change* in dynamics due to change in dose between mouse and human, therefore this scaling the ELISPOT readout was not essential.	

**<u>CD4+ T cell stimulation greatly simplified</u>**<br>The immune response to vaccination is a complex network of cells and cytokines behaving nonlinearly over time. In the Th1 response to *Mtb*. infection (or vaccination), innate and adaptive cells interact to optimise and maintain a protective response [8]. Very simply, cytokines secreted by innate cells after infection or vaccination, such as IL-12, work to stimulate adaptive cells to produce IFN-γ that both encourages innate cells to phagocytose bacteria and produce more IL-12 [9,10]. As such, a feedback stimulation loop is established. In addition, to avoid an over-inflammatory response (which is harmful to the host) cytokines such as IL-10 are produced to regulate and dampen the immune response [11]. In the model, function δ is used to represent the delay of T cell initiation due to processes such as antigen processing and presentation and the decline of T cell responses due to depreciation of the required stimulation (creating a "n-shaped" curve). However, δ neglects the influence of stimulation amplification as a result of cytokine feedback loops, amongst other co-stimulation factors. As such, δ is a generalization of the complex networks required to protect against infection or vaccination and may not be as prolonged as required to generate a response to vaccination.	If data were available on IL-12 or other cytokines believed to be important to an immune response to BCG, It is possible that δ could be modelled as a parallel "innate response" compartmental model. Incorporating such a model would provide insight into the innate cell mechanisms and thus strengthen the conclusions we draw on the T cell dynamics.
**<u>Transition and replication of transitional effector cells happens in Lymph node before entering the blood</u>**<br>The model assumes that the recruited transitional effector cells are former *Mtb*.-specific naïve CD4+ T cells that have clonally expanded within the *lymph node*	To incorporate replication of transitional effector cells into the model, a parameter R_E would be applied which would determine the rate at which replication

and exited into the blood stream. Under this assumption, transitional effector cells do not replicate in this model. The rate of naïve CD4+ T cell clonal expansion changes with time dependent on stimulation from innate processes and antigen presence [7] so could be considered to be incorporated into δ.	occurs, dependent on the current transitional effector cell count.

Table S3. Main assumptions of the model and implications on challenging these assumptions

Additional Methods

Data

Mouse IFN-γ ELISPOT data

The methods and materials used to generate the mouse IFN-γ response data following H56+IC31 vaccination are outlined below. These methods are published in [12].

Ethics Statement

All animal work was carried out in accordance with the Animals (Scientific Procedures) Act 1986 under a license granted by the UK Home Office (PPL 70/8043), and approved by the LSHTM Animal Welfare and Ethics Review Body.

Animals

Female CB6F1 mice were acquired from Charles River UK at 6-8 weeks of age. Animals were housed in specific pathogen-free individually vented cages, were fed ad libitum, and were allowed to acclimatize for at least 5 days before the start of any experimental procedure.

Vaccination

The experimental vaccine H56 (comprising *Mycobacteria tuberculosis* antigens Ag85B-ESAT-6-Rv2660c [13], provided by Statens Serum Institute (SSI), Copenhagen, Denmark) was formulated in IC31® adjuvant (provided by SSI on behalf of Valneva Austria GmbH) and 10 mM Tris-HCL buffer (pH 7.4) as described in [14] to obtain a final volume of 200 µl/mouse. The adjuvant IC31®consists of a mixture of the cationic peptide KLK (NH2-KLKL5KLK-COOH) and the oligodeoxynucleotide ODN1a (oligo-(dIdC)13). Adjuvant doses were 100 nmol peptide and 4 nmol oligonucleotide for every vaccine (H56) dose. Antigen doses of 0.1, 0.5, 1, 5 or 15 µg of H56 + 100/4 nmol IC31 (hereafter, H56+IC31) were administered per animal at day 0 and 15, the same dose was used at both vaccination times within a group. Control animals received no vaccination. The vaccine was administered subcutaneously into the left or right leg flap. Animals were not randomized and no blinding was applied.

IFN-γ ELISPOT

IFN-γ secreting CD4+ T cells were measured using the ELISPOT assay. Single cell suspensions of mouse splenocytes were prepared by mechanical disruption of spleens throμgh a 100μm cell strainer on the day of sacrifice. After lysis of red blood cells, single cell suspensions were made up in antibiotic-free media [RPMI-1640 (Sigma-Aldrich, Dorset, UK) + 10% heat-inactivated FBS (Labtech International Ltd, Uckfield, UK) + 2 mM L-Glutamine (Fisher Scientific, Loughboroμgh, UK)]. 96-well microtiter ELISPOT plates (MAIPS4510, Millipore, Watford, UK) were coated with 10 μg/ml rat anti-mouse IFN-γ (clone AN18, Mabtech, Nacka Strand, Sweden). Free binding sites were blocked with RMPI 1640 supplemented as described above. $2.5x10^5$ of total splenocytes were added and incubated in duplicate with H56 (10 μg/ml), supplemented RPMI as a negative control, or Phorbol myristate acetate (PMA) (50 μg/ml, Sigma-Aldrich) and Phytohemagglutinin (PHA) (10 μg/ml, Sigma-Aldrich) as a positive control. After 24 or 48 hrs of incubation at 37°C in 5% CO_2, IFN-γ was detected with 1 μg/ml biotin labelled rat anti-mouse antibody (clone R4-6A2, Mabtech) and 1 μg/ml alkaline phosphatase-conjugated streptavidin (Mabtech). The enzyme reaction was developed with BCIP/NBT substrate (5-Bromo-4-chloro-3-indolyl phosphate/Nitro blue tetrazolium) (MP Biochemicals, UK) and stopped by washing the plates with tap water when individual spots could be visually detected (up to 5min). ELISPOT plates were analysed using an automatic plate reader. IFN-γ-specific cells are expressed as number of spot-forming units (SFU) per million spleen cells after non-specific background was subtracted using negative control wells.

Experimental Schedule

ELISPOTs were carried out at 2, 7, 9, 14, 16, 21, 28, and 56 days after the first vaccination for all doses. Five mice were used per time point per dose group (equating to 40 mice in a dose group from initiation to conclusion of the experiment). This schedule was designed to reflect the H56+IC31 phase I clinical trial schedule [15] and previous experiments and schedules in mice using the H-series vaccines by SSI in CB6F1 mice [13,16-18].

Figure S11 shows the ELISPOT results using the 24 hour incubation time for each dosing group. Each coloured dot represents the responses of one mouse, the lines indicate the median responses.

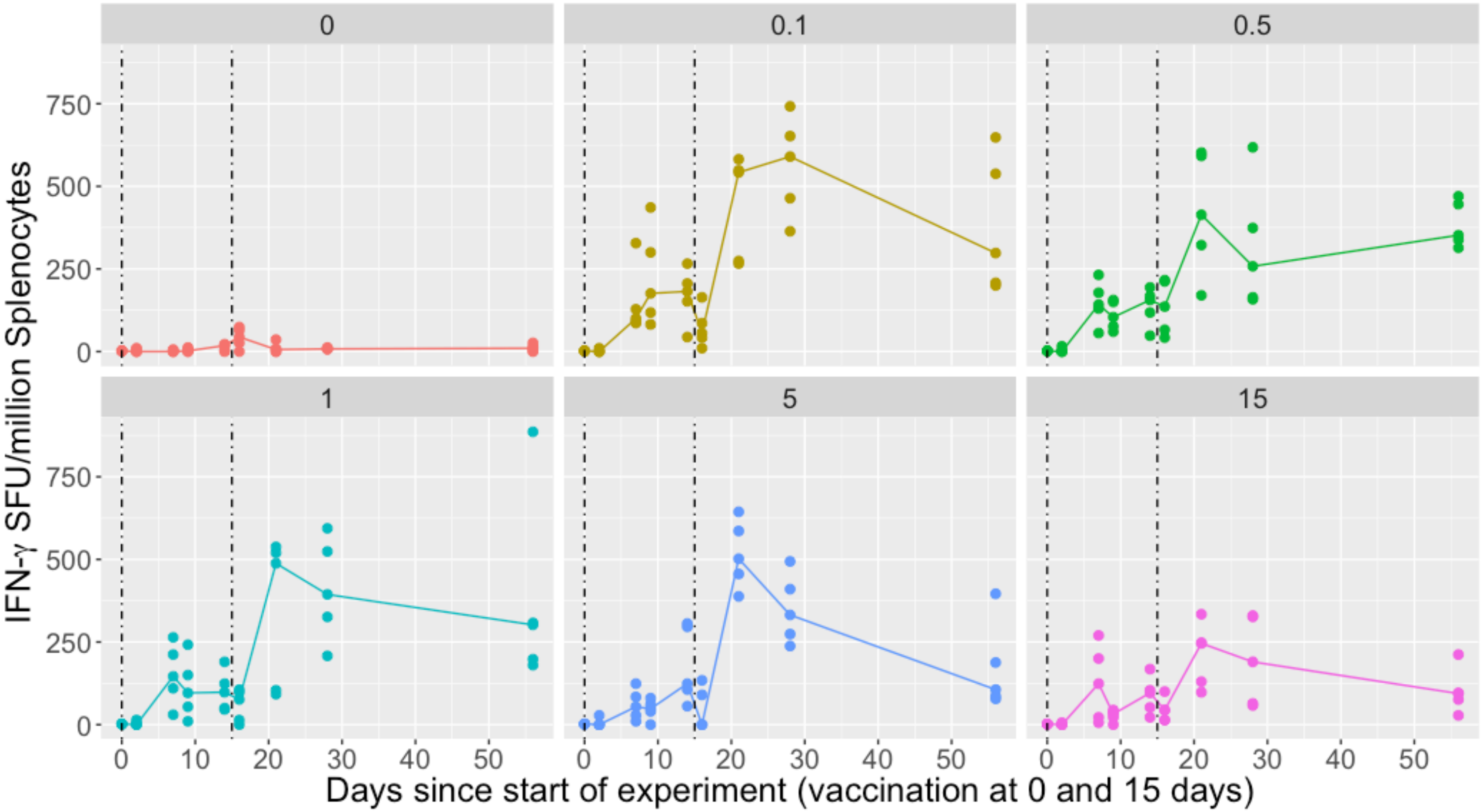

Figure S11. Median IFN-γ responses (lines) and responses of individual mice per time point (coloured points) for each dose including control dose. Panel titles refer to H56 antigen dose, IC31 was 100nmol throughout. Dashed vertical lines correspond to vaccination times.

Human IFN-γ ELISPOT data

Table S4 summarizes the two H-series trials from which the human ELISPOT data was taken. Figure S12 shows the individual IFN-γ responses (measured using ELISPOT assay) over time for both trials and the pooled median response across both trials.

	Clinical trial Information					Data from Clinical trial used in the analysis					
Vaccine	ClinicalTrials.gov ID/publication	Phase	Purpose of trial (taken from ClinicalTrials.gov)	Country conducted	Study arms	Study arm used	N	Response measurement times (days)	Median age (IQR)	Gender	Years since BCG
H56+IC31	NCT01967134/[15]	i	Evaluation of the Safety and immunogenicity profile of H56+IC31 administered to HIV-negative adults and without LTBI and no history or evidence of tuberculosis (TB) disease.	South Africa	1. N=8, LTBI negative, dose = 50 µg H56(+500 nmol IC31), two vaccinations (day 0, 56)<br>2. N=8, LTBI positive, dose = 15 µg H56(+500 nmol IC31), two vaccinations (day 0, 56)<br>3. N=9, LTBI positive, dose = 50 µg H56(+500 nmol IC31), two vaccinations (day 0, 56)	1	8	0, 14, 56, 70, 112	32 (19–38)	M=4, F=4	>10 (assumed to be vaccinated at birth)
H1+IC31	NCT00929396/[19]	i	A safety and immunogenicity Phase 1 Trial with an adjuvanted TB subunit vaccine H1+IC31 (Ag85B-ESAT-6 + IC31) administered in PPD positive volunteers at 0 and 2 months	Netherlands	1. N=10, LTBI negative, BCG positive, dose= 50 µg H1(+500 nmol IC31), two vaccinations (day 0, 56)<br>2. N=10, LTBI positive, dose= 50 µg H1(+500 nmol IC31), two vaccinations (day 0, 56)	1	10	0, 7, 42, 63, 98, 224	49 (24–54)	M=7, F=3	>2

Table S4. Outline of the H56+IC31 and H1+IC31 phase i clinical trials and human demographics for each. Abbreviations: LTBI = Latent Tuberculosis Infection, IQR= Inter quartile range.

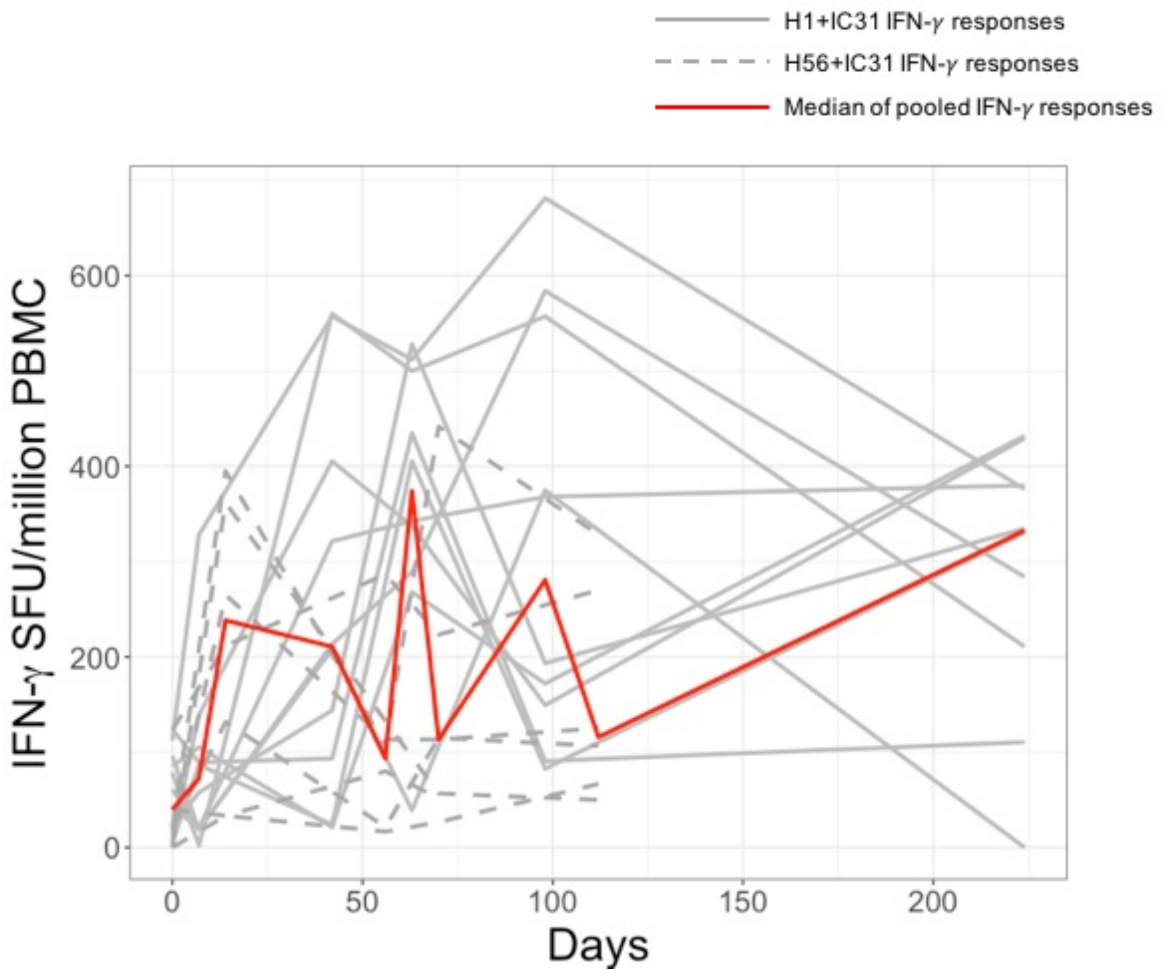

Figure S12. Number of IFN-γ secreting CD4+ T cells in humans in H56+IC31 phase I trial [15] (ClinicalTrials.gov no NCT01967134) and H1+IC31 phase I trial [19] (ClinicalTrials.gov no NCT00929396) over time measured using an ELISPOT assay. Vaccinations of the respective vaccines were given at day 0 and day 56. The median of the pooled data is shown in red and the responses in those that received H1+IC31 are shown in solid grey and for those that received H56+IC31 are shown in dashed grey.

The committees approving the study protocol were: The Medicines Control Council of South Africa and the Human Research Ethics Committee of the University of Cape Town for [15] and the accredited Ethical Review Board of LUMC and the relevant national authorities for [19]. Informed consent was obtained from all participants, as stated in [15] and [19].

Laboratory procedures for the human IFN-γ data

H56+IC31 phase I trial [15]: ClinicalTrials.gov no NCT01967134

Screening procedures for HIV status included a medical history and blood collection for baseline chemistry. QuantiFERON®-TB Gold In Tube test (qft, cellestis limited) was used to determine latent TB infection (LTBI) status.

H1+IC31 phase I trial [19]: ClinicalTrials.gov no NCT00929396

The ELISPOT methods for the H1+IC31 clinical trial are outlined in [19]. We summarise the methods below.

Frozen cells were pre-stimulated for 16-18 hours, followed by 24 hours in the ELISPOT plate. $1x10^6$ thawed cells/well were stimulated in 24 well plates with H1 antigens (Ag85B and ESAT-6 proteins) as well as PPD, separate peptide pools and positive and negative controls (see [19]). All samples were assayed in triplicate. Incubation was done overnight in a fully humidified incubator at 37 ◦C, 5% CO2. Subsequently, cells were resuspended and divided over 3 wells (250,000 cells/well) of a mixed cellulose ester-backed 96 well plate (MAHAS45, Millipore) which had been pre-coated with anti-IFN--antibody (mAb1-D1K, Mabtech, Sweden) and blocked with AIMV medium. The next day biotinylated detector antibody (mAb 7-B6-1, Mabtech) was added and spots colored with alkaline phosphatase conjµgated streptavidin (Mabtech, Sweden) and FastTMNBT/BCIP (Sigma–Aldrich, The Netherlands). Substrate incubation was done at room temperature for 10 min and stopped by rinsing the plates with tap water. Plates were dried and spots were counted in the Bioreader 3000 pro (BioSys, Germany) using calibrated parameters.

BCG vaccination status was determined by tuberculin-skin-test (TST), whereby a reaction range 6–15mm or any documented value between 6 and 15mm on medical file in the past, indicated the participant was BCG vaccinated. To determine LTBI status, a QuantiFERON®-TB Gold In Tube test and a 6-day lymphocyte stimulation test (as described in [20]) in addition to chest X-rays, were conducted at screening. HIV status was determined by reviewing recorded medical history and conducting standard blood tests.

Mathematical vaccine Immunostimulation/Immunodynamic (IS/ID) Model

The conceptual mechanisms of the IS/ID model for the IFN-γ secreting CD4 T cells as a result of H56+IC31 vaccination can be found in Figure 1. The mathematical model used in Monolix to estimate the parameters represents these exact mechanism, however we separated the compartments into TEM_1 and CM_1 and TEM_2 and CM_2 corresponding to TEM and CM after primary vaccination and TEM and CM after revaccination. Here, once primary vaccination occurs, cells are recruited at rate $\delta_{time=0}$ into TEM_1 and these cells then either die (at rate μ_{TEM}) or transition to CM_1 at rate β_{TEM}. At time of revaccination two process are initiated simultaneously. The first is recruitment into TEM_2 at rate $\delta_{time=revacc}$ (this is the same recruitment, but adjusted for revaccination time; parameter values are the same as in the case of primary vaccination). The second is the replication of CM_1 cells. After τ days, the CM_1 cells stop replicating and transition to TEM_2 at rate β_{CM} which is fixed to an arbitrarily high value so that this process is instantaneous. TEM_2 cells then either die (at rate μ_{TEM}) or transition to CM_2 at rate β_{TEM}. The output of the model is the sum of all TEM and CM compartments over time (see main methods for justification of this).

In summary, this model essentially separates the dynamics of the cells corresponding to which vaccination time they follow, but retains the overall conceptual mechanisms outlined in Figure 1. The main reason for doing this was to eliminate the risk of cells continually transitioning back to TEM (as a result of the parameter β_{CM}) as may occur if an exact model of that outlined in Figure 1 was adopted. In the conceptual schema in Figure 1, the only way to eliminate this from happening was to apply a condition on β_{CM} to be 0 once 99% of CM cells had left the CM compartment after replication. This would have added complexity to the Monolix code we felt was unnecessary. We felt the representation of the model dynamics in

Figure 1 was easier for a reader to understand conceptually than the model of the separated compartments.

As such, the equations for the model are as follows:

$$\frac{dTEM_1}{dt} = \delta_{time=0} - \beta_{TEM}TEM_1 - \mu_{TEM}TEM_1 \quad (1)$$

$$\frac{dCM1}{dt} = \beta_{TEM}TEM_1 + R_{CM}CM_1 - \beta_{CM}CM_1 \quad (2)$$

$$\frac{dTEM2}{dt} = \delta_{time=revacc} - \beta_{TEM}TEM_2 - \mu_{TEM}TEM_2 + \beta_{CM}CM_1 \quad (3)$$

$$\frac{dCM2}{dt} = \beta_{TEM}TEM_2 \quad (4)$$

Where t, the time in days and the parameters are those outlined in Figure 1. The parameters in the model follow the rules:

- δ initiated at time=0 and time=revaccination and has the same parameter values at both times.
- R_{CM} = 0 until time = time of revaccination then 0 after time = time of revaccination + τ
- $\beta_{CM} = 0$ until time = time of revaccination + τ. The value of β_{CM} is fixed arbitrarily high, at a value of 10 cells per day.

A schema of the model is below.

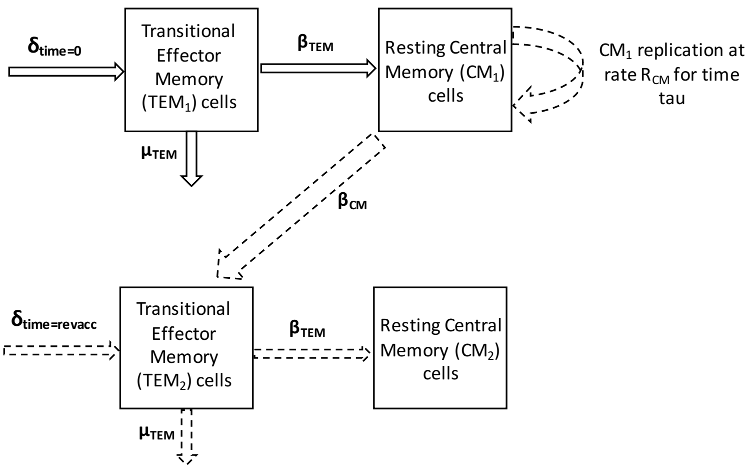

Where dotted arrows correspond to rates activated at time of (or following in the case of β_{CM}) revaccination.

Model Fitting

Statistical (NLMEM) model

In this work, we use the method of Nonlinear Mixed Effects Modelling (NLMEM) to estimate the free model parameters. NLMEM is a statistical framework which combines a mathematical or statistical model to describe the longitudinal response data over time and statistical models to capture variation in the mathematical model parameters due to multiple individual responses in a population. Using NLMEM inferences can be made about the variation in response across a population when population covariate analysis is conducted [21,22].

The main aims of NLMEM are [23]:

1. To estimate the free parameters of the mathematical model that describe the population typical response dynamics over time
2. Estimate the variation around the population average dynamics as a result of individuals in the population (inter-individual variation) as thus estimate the individual responses
3. Establish residual variation between model prediction and response data (intra-individual variation)
4. Assess the effect of population covariates on the population typical dynamics (mathematical model parameters) and associated variation (statistical model parameters)

To conduct NLMEM to estimate the IS/ID model parameters for the H56 IFN-γ response data for mice and humans, the following is required:

1. Statistical models to account for individual responses.

To account for the intra and inter- variation, the NLMEM framework statistical model requires two components:

1. The proposed distribution of the free model parameters from the population typical response to account for individual responses over time. In this work, we assume the individual parameters are lognormally distributed around the population typical value.
2. The relationship between the error of the data from the mathematical model prediction, known as the residual error, on an individual level of time (assumed to be the same distribution for each individual). The residual error (RE) model is applied to account for discrepancies between the observed data and the model prediction. The assumption in NLMEM is that the REs are normally distributed, but may be dependent on the magnitude of the response (i.e. it is intuitive that the error may be higher for higher values of the response than for lower values). Three common models are outlined in the table below. We only concentrate on these three models in our fitting as they are generally considered to reflect lab assay variability patterns [24]. Alternatives are outlined in [24].

Error model	Model equation	Description
Constant	Y = f+a*e	Constant residual error variance from the model prediction, f
Proportional	Y = f+b*f*e	Proportional residual error variance when we believe the variance is proportional to the model prediction, f
Combined	Y = f+(a+b*f)*e	A combination of constant and proportional residual error variances

where Y = data point, f = model prediction, a,b= scalars to be determined during parameter estimation process, e = Normally distributed random variable N(0,1).

2. A covariate model structure.

A way of incorporating population covariates to establish if parameter estimates are significantly different between subpopulations and what these values are. In this work, the parameter-covariate relationship was multiplicative, for example, the population estimation of the transition from transitional effector memory cells to central memory cells, β_{TEM}, in accounting for dose group was modelled by $\beta_{TEM:Middle}=\beta_{TEM:Low}*e^{\alpha m}$, where $\beta_{TEM:Middle}$ is the

value for β_{TEM} for those mice that were in the middle dose group (the reference group) and αm is the exponentiated scalar of this value to represent changes in β_{TEM} for those in the low dose group. For those mice in the high group this was $\beta_{TEM1:Middle}=\beta_{TEM1:High}*e^{\alpha l}$. The covariate effects (α's) are estimated in the NLMEM analysis alongside the associated p-values, but the value for the group parameter (left hand side of above equation) is reported in the results.

Diagnostic Plots

a. *Visual Predictive Check (VPC) plot*

   The visual predictive check plot (VPC) is a simulation based diagnostic tool for assessing the appropriateness of the proposed mathematical model to describe the empirical data. This is done by comparing data simulated using the model and estimated population mean parameters and associated variances, to the empirical data distribution [23]. To construct the VPC, the mathematical model is fitted to the dataset in question and the resulting estimated parameters and associated variances are used to simulate a theoretical population dataset, equivalent to the size of the population in question. This procedure is repeated 500 times and key percentiles (e.g. the 25th, 50th and 75th percentiles) of each simulated population dataset are recorded and the ranges of these percentiles are plotted. If the model is appropriate to represent the data, when the observed percentiles are plotted alongside the VPC, they should fall in the bounds of the simulated percentile ranges. These plots are common as a diagnostic tool in PK/PD modelling and have been suggested as the most efficient plots to assess a NLMEM parameter estimation [25]. The plots are produced by Monolix as a standard output.

b. *Parameter prediction plot*

   The parameter prediction plots show the prediction of the mathematical model for the estimated population parameters compared to the median of the data (the population typical response) and the distribution of the model predictions due to the estimated parameter variation (to cover the population spread of the data, usually the 10th to 90th percentile or the 25th to 75th). Plots included in the work are either Monolix standard outputs (those with pink percentile bands).

c. *Observed versus predicted data plots*

In these plots, the observed data is plotted against the predicted value on a population and individual level. Plots where the observed and predicted values are similar should show a diagonal distribution along a line of unity (i.e. if the prediction is the same as observed value that point will lie on the diagonal line extending from the origin of the plot).

Monolix code

Alongside the data, which is outlined in figures S11 and S12, the statistical model outlined in the methods of the main paper, the monolix code used to fit the IS/ID model to the data can be found below:

```
<MODEL>
DESCRIPTION:
[LONGITUDINAL]
INPUT:
parameter = [Death,TransR,a,b,c,TCM] ;;change to curly brackets
EQUATION:
t0 = 0   ;;Time initial condition
E_0=1 ;;TEM1 initial condition (value of 1 is median mouse baseline response, 40 for humans)
M_0=0 ;;CM1 initial condition
E2_0=0 ;;TEM2 initial condition
M2_0=0 ;;CM2 initial condition
;;;;;;;;;;;;;Parameters in the model equations
   m=1/Death ;;death rate of TEM cells
   Btem=1/TransR
   Rmem=1.8
    BCM=10

d1 = a*exp((-(t-b)^2)/(2*c^2)) ;;;delta parameter for first vaccination
dR = a*exp((-(t-(16+b))^2)/(2*c^2)) ;;;delta parameter for first vaccination (adjusted for revaccination time)
;;;;;;;;;;;;;;;;;;;;;;;;;Conditions on CM cells to replicate due to revaccination
    if t>=(16)  & t<=(16+TCM)     ;;CM1 cells replicate for TCM time
      Rm = Rmem
else
      Rm = 0
```

```
End

 if t>=(16+TCM)      ;;CM1 cells transition to TEM2 type after replication
     B = BCM
 else
     B = 0
End

;;;;;;; Equations
   ddt_E =d1 - Btem*E - m*E            ;;TEM1 dynamics
   ddt_M = Btem*E-B*M+Rm*M     ;;CM1 dynamics

   ddt_E2 = dR - Btem*E2 - m*E2+B*M          ;;TEM2 dynamics
   ddt_M2 = Btem*E2        ;;CM2 dynamics

 Total=E+M+E2+M2 ;;TEM1 and 2 and CM1 and 2 are summed to give the total over time
OUTPUT:
output = Total
```

Justification for fixing the random effects due to between subject variability (BSV)

Given the sample size of the data and what we aimed to achieve (using the model to describe the IFN-γ dynamics by dose), we prioritised estimating the "average" response for as many subpopulations (dose groupings) as possible (the population typical model parameter values for each dose group), over estimating variation in response for those subpopulations (the BSV of the model parameters).

As such we implemented the following tasks until power to achieve well estimated parameters was lost due to sample size:

For the mice

1. Fit the model to the population pooled data and achieve well estimated (RSE<30%) population typical parameter values, with parameter standard deviations to account for BSV=0.5.

2. Fit the model to the mouse data stratified by dose with one model parameter indexed on dose (see NLMEM technical description for example of indexing), with parameter standard deviations to account for BSV=0.5.
3. Continue to index more model parameters on does group until sample restricts estimation precision.
4. Estimate the parameter standard deviations to account for BSV.

For humans:

1. Fit the model to the population pooled data and achieve well estimated (RSE<30%) population typical parameter values, with parameter standard deviations to account for BSV=0.5.
2. Estimate the parameter standard deviations to account for BSV.

Testing the structural model for parameter δ and fit of the IS/ID model to the pooled mouse data

Three different mathematical forms were used to represent parameter δ:

1. Gamma Probability Density Function (PDF) equation:

$$\delta = L * \frac{\left(1/h\right)^{k}}{\Gamma(k)} * time^{(k-1)} * e^{-\left(\frac{1}{h} * time\right)}$$

   the parameters were: *L*=multiplier to scale up the gamma PDF, *k*= Gamma PDF shape parameter, *h*=Gamma PDF scale parameter.

2. Gaussian function equation:

$$\delta = a * exp \frac{-(time - b)^2}{2c^2}$$

   the parameters were: *a*=height of Gaussian function, *b*=mean of Gaussian function, *c*=variance of Gaussian function.

3. Naïve T cell compartment: A naïve T cell compartment was added to the model which introduced cells to the Transitional Effector Memory (TEM) compartment. There were initially 10 cells in the naïve compartment, which replicated every 10 hours for τ_N days. After this, they left the Naïve compartment and enter the TEM compartment at

rate β_N. As naïve cells do not express cytokines until they are differentiated [7], they do not contribute to IFN-γ output of the model. They were also long-lived cells [26], so do not die.

These delta equations initiate at time 0 and time 15 to reflect the two vaccination times.

Table S5 shows the result of the fit of the model to the pooled mouse data for each form of δ in the model. The Gaussian equation provided the best fit according to the BIC value and all parameters were well estimated (RSE<30%).

Form of δ	Model parameters (fixed or to be estimated in Monolix)		Results after fit to pooled mouse data		
	Fixed (value)	To be estimated	Parameters with RSE >30%	BIC value	-2LL value
Gamma PDF	μ_{TEM} (0.3 day^{-1})*, β_{CM} (10 per day^{-1})$^{\&}$	L, k, h, β_{TEM}, τ	None	2453	2415
Gaussian equation		a, b, c, β_{TEM}, τ	None	2382	2344
Naïve compartment		τ_N, β_N, β_{TEM}, τ	None	2503	2471

Table S5. Results of fitting the model to the pooled mouse data for the three forms of δ. *Fixed to value found in literature, &Fixed to assumed high value, 10 cells per day

As outlined in the main paper: the Gaussian equation is as follows (Figure 1, Table S5):

$$a * \left(e^{\frac{-(t-b)^2}{2c^2}} + e^{\frac{-(t-(b+t\_r))^2}{2c^2}} \right)$$

where *a* is a scalar, *b,* the Gaussian equation mean, *c*, the variance, t is *time*,measured in days and t_r is revaccination time measured in days. The estimated parameter values for the Gaussian equation fit to the pooled mouse data are in Table S6.

	**Pooled mouse data**	
**IS/ID Model (figure 1) parameter description (unit)**	**Parameter Value**	**RSE (%)**
Death rate of Transitional effector memory cells, μ_{TEM} (per day)	0.3 (NE)*	-
Transition rate from Transitional Effector to Central Memory cell type, β_{TEM} (per day)	0.18 (E)	17
Replication rate of Central Memory cells (per day), R_{CM}	0.4 (NE)**	-

Central Memory cell replication time, τ (days)	1.24 (E)	14
Transition rate from Central Memory to Transitional Effector type, β_{CM} (per day)	10 (NE)$	-
Recruitment of Transitional Effector rate δ: Gaussian equation scalar, a (# cells)	92 (E)	14
Recruitment of Transitional Effector rate δ: Gaussian equation mean, b (days)	6 (E)	8
Recruitment of Transitional Effector rate δ: Gaussian equation variance, c (days)	1.15 (E)	15

Table S6. Population parameters for the pooled mouse data. Parameters estimated using the Nonlinear Mixed Effects Modelling (NLMEM) framework are indicated with an (E). Those that were not estimated (fixed to a value found in literature or under a model assumption) are indicated with an (NE), their values come from the following sources/assumptions: *[27], **[28] $ Fixed to be high, at a value of 10 cells per day. All estimated model parameter standard deviations were fixed at 0.5. Abbreviations: RSE = relative standard error.

The model predicted IFN-γ responses for the parameter set in table S6 are plotted in Figure S13. The visual predictive check (VPC) plot showed the model predictions represented the median pooled data well (Figure S14).

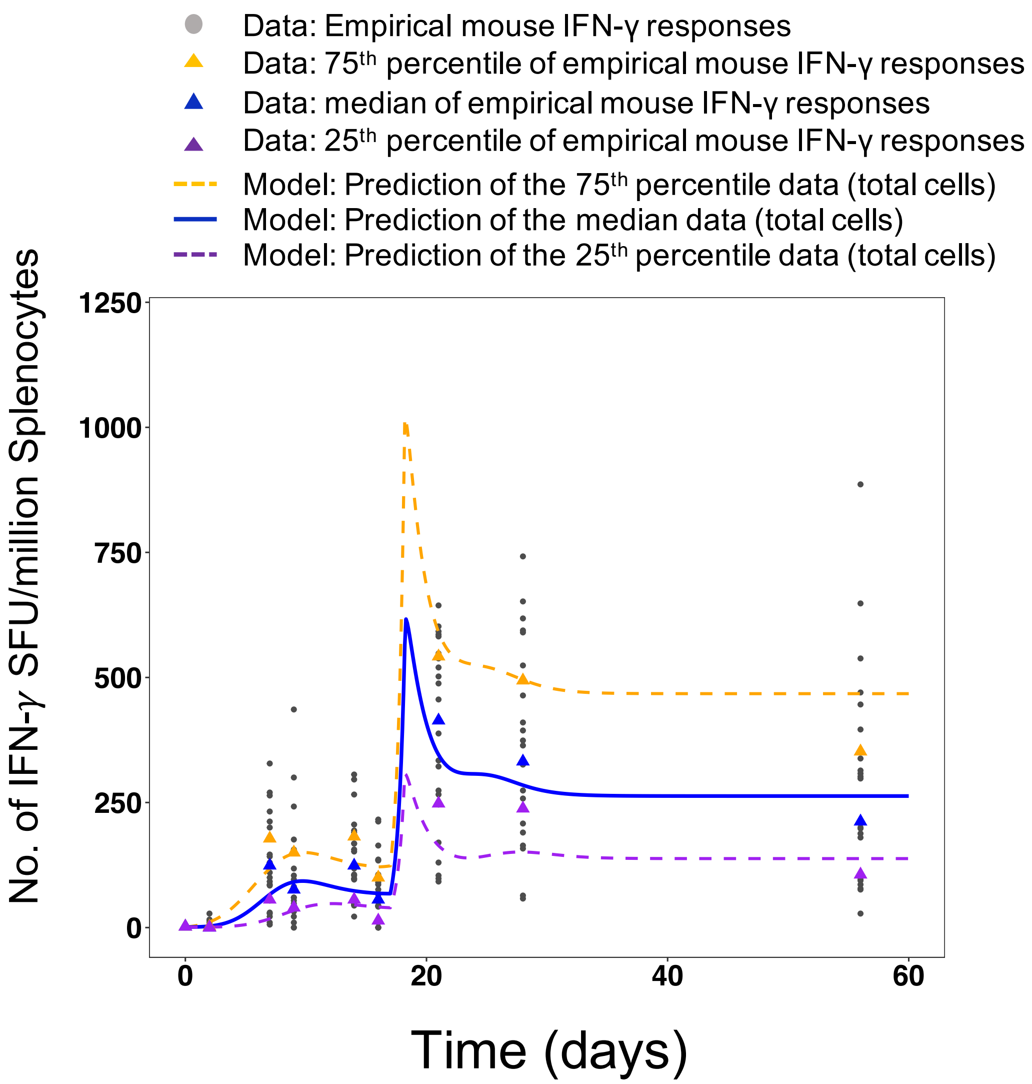

Figure S13. Empirical and model predicted number of IFN-γ secreting CD4+ T cells over time for the pooled mouse data. Grey points correspond to number of IFN-γ secreting CD4+ T cells measured over time by ELISPOT assay in mouse splenocytes for each mouse after receiving vaccination of H56+IC31 at day 0 and day 15. Median responses over time are marked by a blue triangle, the 75th percentile responses by an orange triangle and the 25th percentile responses by a purple triangle. The model prediction (total cells) fitted to the data in the fitting framework (parameters in Table S6) is plotted against the median data (blue line). The orange and purple dashed lines are the model prediction (total cells) of the 75th and 25th percentiles of the data, a result of the variation in the estimated parameters (standard deviation fixed to 0.5 for all parameters (Table S6)).

The VPC plot shows that the simulated model predictions cover the data well and there are little red areas (red areas indicate the simulated model predictions did not adequately cover the observed data) (Figure S14). The red areas in the early response stages may be due to variable responses at this stage. The red area for the 25th percentile prediction indicates the model is under predicting the data. This could be due to the 0.5 value constraint placed on the standard deviation of the parameters which limit the degree to which the predictions can

vary to cover the data. Allowing the standard deviation of the parameters to be estimated was not conducted until after the covariate model was established in analysis 1i.

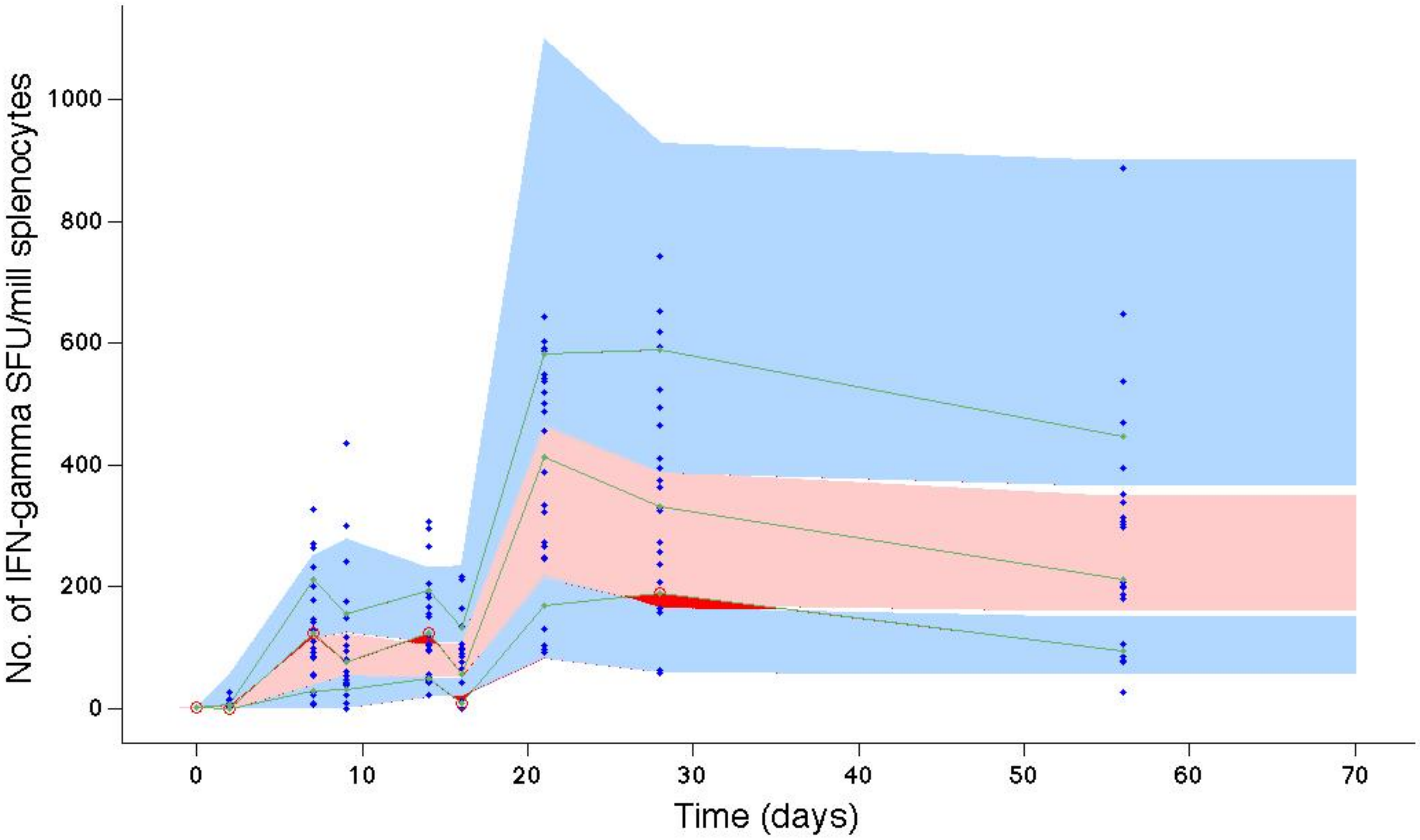

Figure S14. Visual Predictive Check (VPC) plot for the pooled mouse model (parameters from Table S6). Blue points represent the observed data. Blue regions represent the ranges of the 75th and 25th percentiles of the simulated populations. The pink region represents the range of the 50th percentile. The green line links the observed percentiles (25th, 50th and 75th) for each time point. Red regions represent where the observed data falls outside the ranges of the simulated percentiles.

*-2*LogLikelihood sensitivity analysis on the pooled mouse data*

Likelihood sensitivity analysis was conducted by varying one parameter whilst holding the remaining parameters fixed to the values in Table S6 (the fit of the model to the pooled mouse data). The sensitivity range tested was [parameter value/10, parameter value*10] (except for parameter b, which was bound by day 15 to ensure the peak of the recruitment parameter, delta, occurred before revaccination). For the varied parameter, the standard deviation was fixed at 0.5 as a result the lowest -2LL values (those when the parameter is at its best value; those recorded in table S6) are different across the parameters in the sensitivity analysis. The results are plotted and discussed in the figure S15 and table S7 below.

In all cases, the slope of the -2LL sensitivity curves are steeper for the values of the parameters smaller than that of the optimal value (the parameter value where the -2LL is lowest), suggesting that model parameters were fix too low, the description of the data would be likely be worse than if fixed too high.

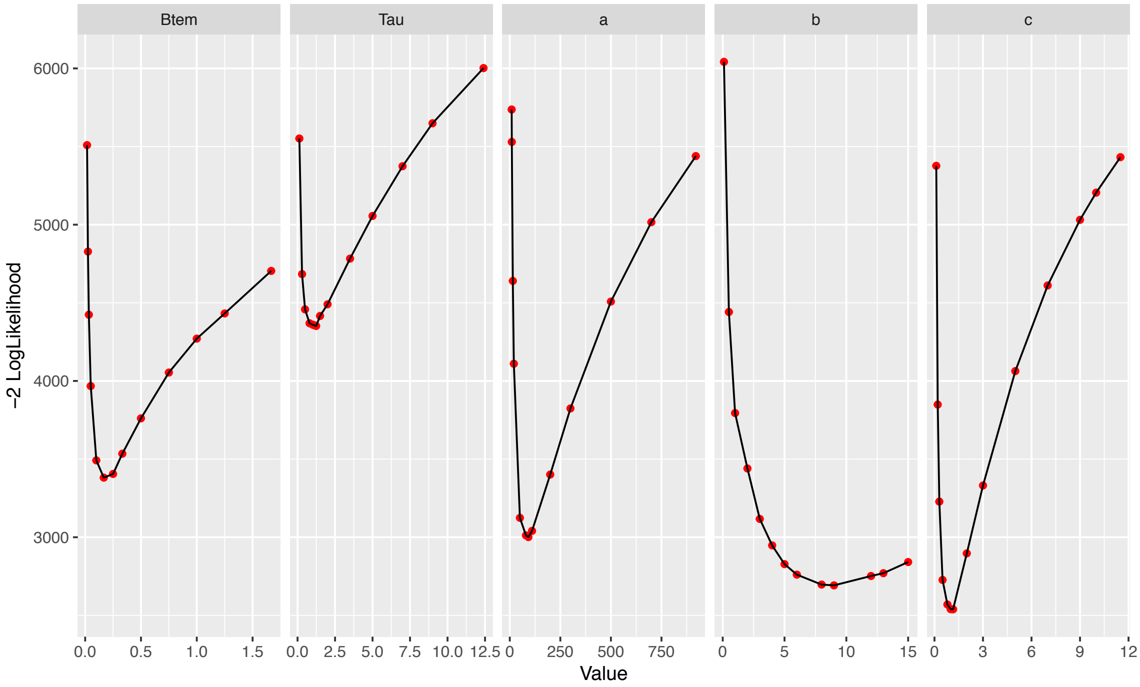

Figure S15. Effects of varying the IS/ID model parameters on the -2*LogLikelihood value (parameter SD=0.5)

Parameter	Comments
c	The likelihood is most sensitive to parameter c in the given parameter range explored (the difference between the maximum and minimum -2LL value (optimal parameter value) is largest for parameter value both below and above the optimal

	value). Parameter c is the variance in the Gaussian equation that describes the TEM recruitment parameter delta. The effect of increasing c on the predicted IFN-γ response over time is a an earlier and more sustained TEM cell recruitment which acts to increase the overall magnitude of response. As such, it is possible that the model is very sensitive to this parameter, so very low and very high values of this parameter will affect the likelihood against all data points (for all times points).
a	The likelihood is sensitive to parameter a in the given range. Parameter a scales the shape of the recruitment rate parameter, delta. An increase in a increases the magnitude of the TEM cell population and therefore the response over all, although unlike the parameter c, a does not regulate the timings of the recruitment only the absolute number of cells. Although similar to parameter c, for the given parameter range in the sensitivity analysis, there is less of a likelihood penalty at larger values of a then for the equivalent change in c.
b	We assumed the parameter b could only take values less than 15, as it was an assumption in the model that the TEM recruitment would be delayed after time of vaccination, but occur before revaccination. Potentially, very early (small) values for this parameter suggest that the response clears too quickly (for all other parameters fixed), which is the likelihood is noticeably worse for smaller values than for larger values (up to day 15).
Tau	The parameter tau represents how long the CM replicate for before they transition back to effector type. For the model output, this dictates how high the height of the peak after vaccination. For all other parameters fixed, this means, too high values of tau will lead to an overall response after revaccination that is too high, the converse is true for too small values of tau. The model is least sensitive to the value of tau. This is potentially as it is the only parameter in the model that only effects the response after revaccination and therefore, only the fit of the model to three time points.
Btem	The result of changing Btem – the transition rate of TEM to CM cells – is the evident in the later time points, before revaccination and in late time points after revaccination. A higher value of Btem suggests more TEM cells are transitioning to CM type, which then contributes to the overall output of the model (as CM cells do not die). This is most apparent for the very late time points of the model as the model output will be 100% CM cells (given a fixed death rate of TEM cells). A lower value of Btem will mean TEM cells may die out before they can transition, lowering the magnitude of response once the initial recruitment of TEM cells has waivered. This is important, as if the CM cells are minimal just before revaccination time, with a fixed Tau, the response may not be boosted high enough to fit the data after revaccination. This may be the reason why the -2LL sensitivity slope increases fast for smaller values of BTem and to higher -2LL values.

Table S7. Discussion on the -2*LogLikelihood sensitivity analysis

Analysis 1: Fitting the IS/ID model to the mouse data stratified by dose group and the human data

Validation of Pooling Human data across Vaccine Type

Table S6 shows the result of indexing vaccine type on the estimated model parameters from the human pooled model.

-2LL value for pooled model	Vaccine type indexed on parameter(s)	Results		Difference in -2LL from pooled model (pooled model-covriate model)	0.01 level significant? (2 or 4 d.f.) (Chi^2 test 2 d.f.: crit val = 9.21, 4 d.f.: crit val = 13.28)
		Parameters with RSE >30%	-2LL		
1237	a	All	1253	-16	(2 d.f.) No<br>-2LL larger
	b	β_{TEM}, τ	1281	-44	(2 d.f.) No<br>-2LL larger
	c	b, β_{TEM}, τ	1241	-4	(2 d.f.) No<br>-2LL larger
	β_{TEM}	b, c	1246	-9	(2 d.f.) No<br>-2LL larger
	τ	b, c, β_{TEM}	1258	-21	(2 d.f.) No<br>-2LL larger

Table S8. Results of indexing the vaccine type covariate on all combinations of estimated parameters in the human pooled model

Table S8 shows that the vaccine type covariate was not associated with a significant improvement in model fit from the model fit to the pooled human data. As all covariate model fits had parameters RSE>30%, we did not test further than one parameter. This result was not surprising as H56 and H1 have been shown to have a similar immunogenicity profile [29]. As

indexing on two model parameters on vaccine type resulted in unidentifiable model fits (for all), we did not analyse the effect of indexing all combinations of three or more model parameters on vaccine type.

References

1 Knudsen, N. P. *et al.* Different human vaccine adjuvants promote distinct antigen-independent immunological signatures tailored to different pathogens. *Sci Rep* **6**, 19570, doi:10.1038/srep19570 (2016).

2 Billeskov, R. *et al.* Testing the H56 Vaccine Delivered in 4 Different Adjuvants as a BCG-Booster in a Non-Human Primate Model of Tuberculosis. *PLoS One* **11**, e0161217, doi:10.1371/journal.pone.0161217 (2016).

3 Reither, K. *et al.* Safety and Immunogenicity of H1/IC31(R), an Adjuvanted TB Subunit Vaccine, in HIV-Infected Adults with CD4+ Lymphocyte Counts Greater than 350 cells/mm3: A Phase II, Multi-Centre, Double-Blind, Randomized, Placebo-Controlled Trial. *PLoS One* **9**, e114602, doi:10.1371/journal.pone.0114602 (2014).

4 Macallan, D. C., Borghans, J. A. & Asquith, B. Human T Cell Memory: A Dynamic View. *Vaccines (Basel)* **5**, doi:10.3390/vaccines5010005 (2017).

5 Macallan, D. C. *et al.* Measurement and modeling of human T cell kinetics. *Eur J Immunol* **33**, 2316-2326, doi:10.1002/eji.200323763 (2003).

6 Homann, D., Teyton, L. & Oldstone, M. B. Differential regulation of antiviral T-cell immunity results in stable CD8+ but declining CD4+ T-cell memory. *Nat Med* **7**, 913-919, doi:10.1038/90950 (2001).

7 Abbas, A., Lichtman, A. & Pillai, S. *Cellular and Molecular Immunology*. 8 edn, (Elsevier Saunders, 2015).

8 O'Garra, A. *et al.* The immune response in tuberculosis. *Annu Rev Immunol* **31**, 475-527, doi:10.1146/annurev-immunol-032712-095939 (2013).

9 Jouanguy, E. *et al.* IL-12 and IFN-gamma in host defense against mycobacteria and salmonella in mice and men. *Curr Opin Immunol* **11**, 346-351 (1999).

10 Flynn, J. L. Immunology of tuberculosis and implications in vaccine development. *Tuberculosis (Edinb)* **84**, 93-101 (2004).

11 Mayer-Barber, K. D. *et al.* Host-directed therapy of tuberculosis based on interleukin-1 and type I interferon crosstalk. *Nature* **511**, 99-103, doi:10.1038/nature13489 (2014).

12 Rhodes, S. J. *et al.* The TB vaccine H56+IC31 dose-response curve is peaked not saturating: Data generation for new mathematical modelling methods to inform vaccine dose decisions. *Vaccine* **34**, 6285-6291, doi:10.1016/j.vaccine.2016.10.060 (2016).

13 Aagaard, C. *et al.* A multistage tuberculosis vaccine that confers efficient protection before and after exposure. *Nat Med* **17**, 189-194, doi:10.1038/nm.2285 (2011).

14 Lalvani, A. *et al.* Rapid detection of Mycobacterium tuberculosis infection by enumeration of antigen-specific T cells. *Am J Respir Crit Care Med* **163**, 824-828, doi:10.1164/ajrccm.163.4.2009100 (2001).

15 Luabeya, A. K. *et al.* First-in-human trial of the post-exposure tuberculosis vaccine H56:IC31 in Mycobacterium tuberculosis infected and non-infected healthy adults. *Vaccine* **33**, 4130-4140, doi:10.1016/j.vaccine.2015.06.051 (2015).

16 Hoang, T. *et al.* ESAT-6 (EsxA) and TB10.4 (EsxH) based vaccines for pre- and post-exposure tuberculosis vaccination. *PLoS One* **8**, e80579, doi:10.1371/journal.pone.0080579 (2013).

17 Aagaard, C. *et al.* Protection and polyfunctional T cells induced by Ag85B-TB10.4/IC31 against Mycobacterium tuberculosis is highly dependent on the antigen dose. *PLoS One* **4**, e5930, doi:10.1371/journal.pone.0005930 (2009).

18 Henriksen-Lacey, M. *et al.* Liposomal cationic charge and antigen adsorption are important properties for the efficient deposition of antigen at the injection site and ability of the vaccine to induce a CMI response. *J Control Release* **145**, 102-108, doi:10.1016/j.jconrel.2010.03.027 (2010).

19 van Dissel, J. T. *et al.* Ag85B-ESAT-6 adjuvanted with IC31(R) promotes strong and long-lived Mycobacterium tuberculosis specific T cell responses in volunteers with previous BCG

vaccination or tuberculosis infection. *Vaccine* **29**, 2100-2109, doi:10.1016/j.vaccine.2010.12.135 (2011).

20 van Dissel, J. T. *et al.* Ag85B-ESAT-6 adjuvanted with IC31 promotes strong and long-lived Mycobacterium tuberculosis specific T cell responses in naive human volunteers. *Vaccine* **28**, 3571-3581, doi:10.1016/j.vaccine.2010.02.094 (2010).

21 Davidian, M. in *Longitudinal Data Analysis* (eds M. Fitzmaurice, M. Davidian, G. Verbeke, & G. Molenberghs) Ch. 5, 107-141 (Chapman & Hall/CRC Press, 2009).

22 Bonate, P. L. in *Pharmacokinetic-Pharmacodynamic Modeling and Simulation* 233-301 (Springer US, 2011).

23 Lavielle, M. *Mixed Effects Models for the Population Approach: Models, Tasks, Methods and Tools*. (Chapman & Hall, 2015).

24 Mould, D. R. & Upton, R. N. Basic concepts in population modeling, simulation, and model-based drug development-part 2: introduction to pharmacokinetic modeling methods. *CPT Pharmacometrics Syst Pharmacol* **2**, e38, doi:10.1038/psp.2013.14 (2013).

25 Holford, N. in *Population Approach Group Europe* (Pamplona, Spain, 2005).

26 Tough, D. F. & Sprent, J. Life span of naive and memory T cells. *Stem Cells* **13**, 242-249, doi:10.1002/stem.5530130305 (1995).

27 Sprent, J. & Basten, A. Circulating T and B lymphocytes of the mouse. II. Lifespan. *Cell Immunol* **7**, 40-59 (1973).

28 Kaech, S. M., Wherry, E. J. & Ahmed, R. Effector and memory T-cell differentiation: implications for vaccine development. *Nat Rev Immunol* **2**, 251-262, doi:10.1038/nri778 (2002).

29 Ruhwald, M., Agger, E. M., Hoff, S. T. & Andersen, P. H1- and H56- subunit vaccines against TB - an overview of the clinical development. *Eur Respir J* **44** (2014).
